# Glutamine exerts a protective effect on osteoarthritis development by inhibiting the Jun N-terminal kinase and nuclear factor kappa-B signaling pathways

**DOI:** 10.1038/s41598-022-16093-7

**Published:** 2022-07-13

**Authors:** Lin Zhong, Le Cao, Rui Song, Xue-Fei Yang, Jia-Le Li, Hai-Tao Yang, Hong-Xiang Zhou, Hai-Tao Fan

**Affiliations:** 1 Department of Orthopedics, The First Affiliated Hospital of Anhui Medical University, #218 Jixi Road, Hefei, 230022 China; 2 Department of Orthopedics, The FuYang Affiliated Hospital of Anhui Medical University, #99 Huangshan Road, FuYang, 236000 China

**Keywords:** Drug discovery, Molecular biology, Zoology

## Abstract

Strategies for treating osteoarthritis (OA) have become a research focus because an effective treatment for OA is unavailable. The objective of this study was to explore the effects and underlying mechanisms of glutamine (Gln) in OA. First, the chondrocytes were identified and a standard IL-1β-induced OA model was established. After treatment with Gln or saline, the viability and apoptosis of chondrocytes were evaluated using a CCK-8 assay and flow cytometry analysis, which revealed that Gln can improve the IL-1β-induced OA cells. Meanwhile, Gln can enhance the expression of aggrecan and collagen II, which are protective proteins for articular cartilage. Instead, Gln inhibited the expression of matrix metalloproteinase-1 (MMP-1) and matrix metalloproteinase-13 (MMP-13), which can degrade cartilage. To better understand the underlying mechanisms of Gln in IL-1β-induced chondrocytes, the classical OA pathways of JNK and NF-κB were examined at the protein and mRNA levels using western blot and qRT–PCR analyses. We found that JNK and NF-κB were downregulated gradually depending on the Gln dose and protective and destructive factors changed based on changes of JNK and NF-κB. The effects of high-dose Gln were more effective than low-dose. Moreover, Gln was applied to the animal OA model to check the effects in vivo. The results showed that Gln attenuated cartilage degeneration and decreased OARSI scores, which demonstrated that Gln can improve OA. The experiments showed that Gln can benefit mice with OA by inhibiting the JNK and NF-κB signaling pathways.

## Introduction

Osteoarthritis (OA) is a common degenerative joint disease without definite treatment that frequently occurs among the elderly population^1^, and it is characterized by articular cartilage erosion and rebuilding, subchondral bone structural change, osteophyte formation, and synovitis^2^. This disease exerts a huge physical and psychological burden on millions of people around the world^3,4^. The clinical symptoms in symptomatic OA patients include joint swelling, pain, disability, and deformity, which affect the patients’ quality of life^5,6^. The etiology involves age, obesity, sex, and heredity; however, the effects of these risk factors on OA at the molecular level have not been clarified. Interleukin-1β (IL-1β) is one of the main initiators in the development of OA, and it exerts harmful effects by upregulating the production of catabolic factors and proinflammatory factors, including nitric oxide (NO), matrix metalloproteinases (MMPs), prostaglandin E2 (PGE2), and thrombospondin motifs (ADAMTS), which ultimately lead to extracellular matrix (ECM) degradation^7–9^. Others have reported that anti-inflammatory treatment can attenuate OA and exert a chondroprotective effect^10^. The essential factor in the progression of OA is an imbalance between anabolic and catabolic components of cartilage, such as MMP1, MMP13, and ADAMTS-5. Accumulating evidence has shown that inflammation and apoptosis play vital roles in the occurrence and progression of OA^11^. Although strenuous efforts have been made to develop a treatment strategy for OA and considerable progress has been observed in trials, satisfactory clinical benefits remain unreachable.

Glutamine is a type of nonessential amino acid that is transported in the plasma and plays an important role in energy metabolism for most cells^12^. It presents several biological characteristics, such as anti-inflammation, antioxidation, and improvement of immunity activities^13–16^. Previous evidence has shown that it significantly inhibits the expression of TNF-α and IL-6 in hepatitis induced by alcohol^17^. Other studies have demonstrated it presents anti-inflammation and antioxidation activity in several inflammatory diseases, which may be related to its ability to suppress the NF-κB signaling pathway^18,19^. Moreover, glutamine has been shown to be the capable of suppressing JNK activity to attenuate TNBS-induced colitis^20^. However, the role of glutamine in OA has not been reported exactly so far.

Nuclear factor kappa-B (NF-κB) is a classical signaling pathway that plays a vital role in inflammation, cellular differentiation, proliferation and survival of normal and malignant cell. Accumulating experimental evidence has shown that NF-κB is an essential factor in the development of OA that promotes articular damage through the induction of COX2, iNOS, and PGE2 and enhances cartilage inflammation and chondrocyte apoptosis^21^, and inhibition of NF-κB can ameliorate proinflammatory responses^22^. JNK is another signaling pathway that has been demonstrated to be associated with the pathogenesis and progression of human OA by many studies^23–26^. Activation of JNK activates c-Jun, a key AP-1 component that leads to decreased proteoglycan synthesis and enhanced cartilage-degrading enzyme MMP-13 production by regulating the expression of proinflammatory cytokines, such as TNF-α and IL-1^27,28^. Accumulated evidence has indicated that the overproduction of MMP-13 by chondrocytes plays a central role in cartilage degeneration^29,30^. Therefore, we hypothesized that Gln exerts anti-inflammatory and cartilage protective effects on OA via the JNK and NF-κB signaling pathways.

## Materials and methods

### Drugs and reagents

Glutamine was purchased from Sigma-Aldrich (St. Louis, MO, USA). Primary antibodies against p-JNK, JNK, p-NF-κB, NF-κB, MMP1, MMP13, ADAMTS-5 and lamin B were provided by CST (Danvers, MA, USA) and Abcam (Cambridge, UK). The FCM assay kits were purchased from Vazyme (Nanjing, Jiangsu, China). A primary antibody against β-actin and all of the secondary antibodies were provided by Affinity Biosciences (Changzhou, Jiangsu, China). Toluidine blue, DAPI and immunofluorescence staining kits were acquired from Beyotime Biotechnology (Shanghai, China).

### Cell extraction, culture, identification, and treatment

Chondrocytes were harvested from 10 OA patients (average age: 63.4 ± 5.8, female: 7, male: 3) who underwent total knee replacement surgery. The chondrocytes were extracted from the normal cartilage that needed to be removed from the knee joint during the operation. The cartilage was cut into small pieces and digested with trypsin for 30 min and 0.1% type II collagenase overnight at 37 °C, and the mixture was transferred into a 10 ml EP tube for centrifugation at 1000 r/min for 5 min. Subsequently, the cell supernatant was resuspended with high-glucose DMEM containing 10% fotal bovine serum and 1% penicillin–streptomycin and cultured in a 37 °C cell incubator. Following three passages, the chondrocytes were used in the experiment. To identify the chondrocytes, toluidine blue and collagen II immunofluorescence staining were employed. After washing three times with PBS, the chondrocytes were fixed with 4% paraformaldehyde for 30 min. Next, the chondrocytes were stained according to the kit manufacturing manuals. Subsequently, the chondrocytes were randomly divided into different groups with different treatments. All human studies received informed consent from the patients and were approved by the ethics committee of the First Affiliated Hospital of Anhui Medical University. The experimental protocol was performed in accordance with the National Institutes of Health (NIH) Guide for the Care and Use of Laboratory.

### Cell viability

The drug toxicity to chondrocytes was verified using an Enhanced Cell Counting Kit-8. Chondrocytes were cultured in 96-well plates for 24 h and then incubated with several different concentrations of Gln (5, 10, 20, 40, or 80 μmol/l) for 24 h. Subsequently, 10 µl CCK-8 was added to each well for 2 h in a 37 °C incubator. Next, the 96-well plates were examined at 450 nm to detect the absorbance in triplicate.

### qRT-PCR

Total RNA was extracted from chondrocytes using TRIzol reagent (Invitrogen, USA) according to the manufacturer’s manual. cDNA was synthesized using a reverse transcription kit (TaKaRa, Tokyo, Japan). The primers were designed and produced by Sangon Biotech Co., Ltd. according to the PubMed Gene Bank (Table 1). qRT-PCR was performed using a 20-μl reaction system for amplification to examine mRNA expression. GAPDH was used as the standard reference. All experiments were performed in triplicate, and the experimental data were analysed by the 2-ΔΔCT method.

**Table 1:** Primer sequences used for qRT–PCR.

Gene	Sequence (5′–3′)	Size (bp)
ADATMS	F:CTTGACGTTCGGGCCTGA R:CACTGTTTCTGGGTGCAG	140
aggrecan	F:CTTCCGCTGGTCAGATGGAC R:CGTTTGTAGGTGGTGGCTGT	189
MMP-1	F:AGAAAGAAGACAAAGGCAAGTTGA R:CCACATCTGGGCTGCTTCAT	147
MMP-13	F:TTGTTGCTGCGCATGAGTTC R:AAGTGGCTTTTGCCGGTGTA	103
JNK	F:CTGAAGCAGAAGCTCCACCA R:CACCTAAAGGAGAGGGCTGC	159
NF-κB	F:CTTCCAAGAAGAGCAGCGTG R:GATCTTGAGCTCGGCAGTGT	160
GAPDH	F:AGTGCCAGCCTCGTCTCATA R:ATGAAGGGGTCGTTGATGGC	133

### WB

Total protein was dissolved and extracted using RIPA lysis buffer (Beyotime, Shanghai, China) supplemented with protease and phosphatase inhibitors. A nuclear cytoplasmic protein extraction kit (Wanleibio, Shenyang, China) was used to extract nuclear and cytoplasmic fractions according to the manufacturer’s instructions. Total protein was measured using a BCA protein assay kit (Beyotime, Shanghai, China) according to the manufacturer’s instructions, and the concentration was uniform. The samples were separated by SDS–PAGE using the same volume per sample. Next, the proteins were transferred to a PVDF membrane (Millipore, MA, USA). After blocking with 5% skimmed milk, the membrane was incubated at 4 °C overnight with primary antibodies against p-JNK (1:1000, CST, 4668S), JNK (1:1000, CST, 9252S), p-NF-κB (1:1000, CST, USA), NF-κB (1:1000, CST, 8242S), MMP1 (1:1000, Abcam, ab137332), MMP13 (1:1000, Abcam, ab39012), ADAMTS-5 (1:200, Abcam, ab41037), aggrecan (1:800, Abcam, ab3773), β-actin (1:2000, Affinity Biosciences, AF7018), and lamin B (1:1000, Abcam, ab16048). The next day, the membrane was incubated with the corresponding secondary antibody after washing with TBST. Following incubation with ECL (Thermo Scientific, USA), the bands were identified with an imaging system (Bio-Rad, USA).

### Flow cytometry (FCM)

To examine the cell apoptosis rate, flow cytometry was employed for the different treatment groups. We used trypsin to digest the cells for 1 min and transferred the mixture to an EP tube. After washing three times with PBS. The precipitate was resuspended with 100 μl 1 × buffer, and then 5 μl annexin V-FITC and 5 μl PI staining solution (Vazyme Biotech, Nanjing, China) were added. Next, the cell apoptosis rate was examined using a flow cytometer after another 400 μl 1 × buffer was added to the mixture.

### Animal study

Eight-week-old C57BL/6 male mice were provided by the Animal Centre of Anhui. Following adaption for one week under a controlled temperature of 24 ± 2 °C and 60 ± 5% humidity, the mice were randomly divided into three groups (n = 5 in each group): sham group, OA group, and Gln group. The animals in the sham group only received a sham operation with a joint capsule incision, and the animals in the OA group were subjected to destabilization of the medial meniscus (DMM). In the Gln group, animals received a DMM operation and an intra-articular injection with glutamine once (100 mM) a week^31^. All animal studies were approved by the Ethics Committee of Anhui Medical University of China and performed in accordance with the protocol in the Institutional Review Board of Anhui Medical University.

### Statistical analysis

All results are presented as the mean ± standard error of the mean (M ± SEM). One-way analysis of variance (ANOVA) with the least significant difference (LSD) and Student-Newman–Keuls (S-NK) tests were used to perform comparisons among multiple groups with SPSS 21.0 (SPSS, Inc., Chicago, IL, USA). The graphs were generated with GraphPad Prism 6.02 (GraphPad Software, Inc., California, USA). A value of p < 0.05 was considered statistically significant.

### Ethical approval

Animal experiments were approved by the Ethics Committee of Anhui Medical University of China (No. LLSC 20211464). All experimental procedures were performed in accordance with the National Institutes of Health (NIH) Guide for the Care and Use of Laboratory.

## Results

### Identification of human primary chondrocytes

**Figure 1:**
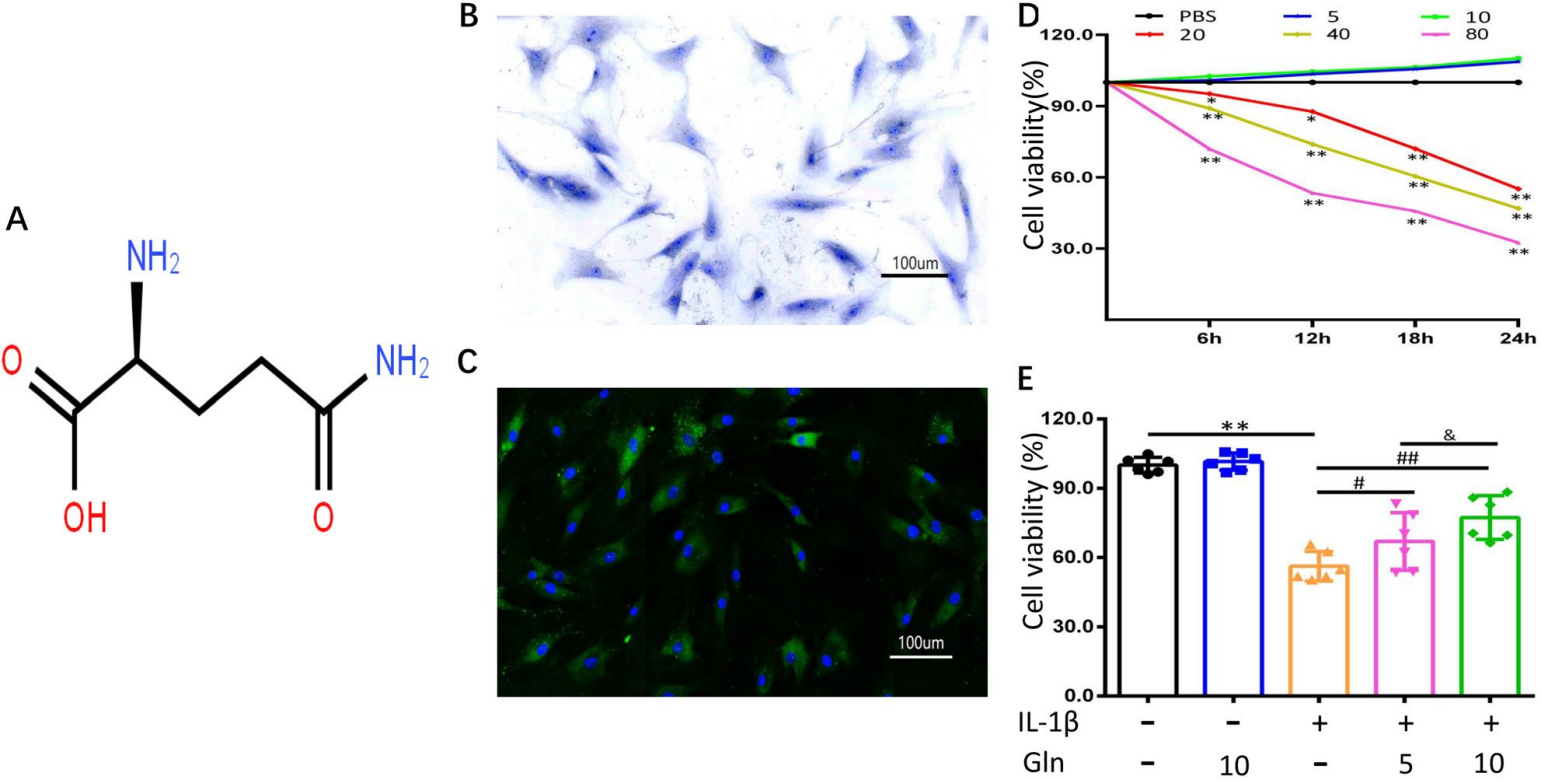
(**A**) Molecular structure of glutamine is shown. (**B**,**C**) Identification of human primary chondrocytes. Picture (**B**) shows the morphological characteristics of human primary chondrocytes using toluidine blue staining. The chondrocytes were stained green by collagen II immunofluorescence staining, and the nuclei were stained blue by DAPI. (**D**) Effect of glutamine on human primary chondrocyte viability. Human primary chondrocytes were cultured in 96-well plates and then treated with different concentrations of glutamine (5, 10, 20, 40, or 80 μmol/l) for 24 h. (**E**) Human primary chondrocytes were pretreated with both doses of glutamine (5, 10 μmol/l) for 2 h and then induced with IL-1β (10 ng/ml) for 24 h. All data are presented as the M ± SEM (n = 6 in each group). *p < 0.05 vs. IL-1β(−) and Gln(−) group, **p < 0.01 vs. IL-1β(−) and Gln(−) group; ^#^p < 0.05 vs. IL-1β(+) and Gln(−) group, ^##^p < 0.01 vs. IL-1β(+) and Gln(−) group; ^&^p < 0.05 vs. IL-1β(+) and Gln(5) group, ^&&^p < 0.01 vs. IL-1β(+) and Gln(5) group by ANOVA.

The molecular structure of glutamine is shown in Fig. 1A. To confirm the identification of primary human chondrocytes, the cells were stained with toluidine blue dye. The results showed that the isolated cells were chondrocytes (Fig. 1B). In addition, collagen II staining revealed that chondrocytes expressed collagen II, which is a typical marker of chondrocytes (Fig. 1C). Overall, these results suggested that human chondrocytes were successfully isolated and identified.

### Effect of Gln on chondrocyte cytotoxicity

To examine the cytotoxicity of Gln to chondrocytes. We used different concentrations of Gln to treat the chondrocytes for 24 h, and cell viability was checked by CCK-8 assay kits. The results showed that Gln (5 and 10 µmol/l) did not lead to significant cytotoxicity for 24 h. However, a higher dose of Gln (> 20 µmol/l) decreased the viability of chondrocytes (Fig. 1D).

Gln affects the viability and apoptosis of human chondrocytes following IL-1β treatment.

**Figure 2:**
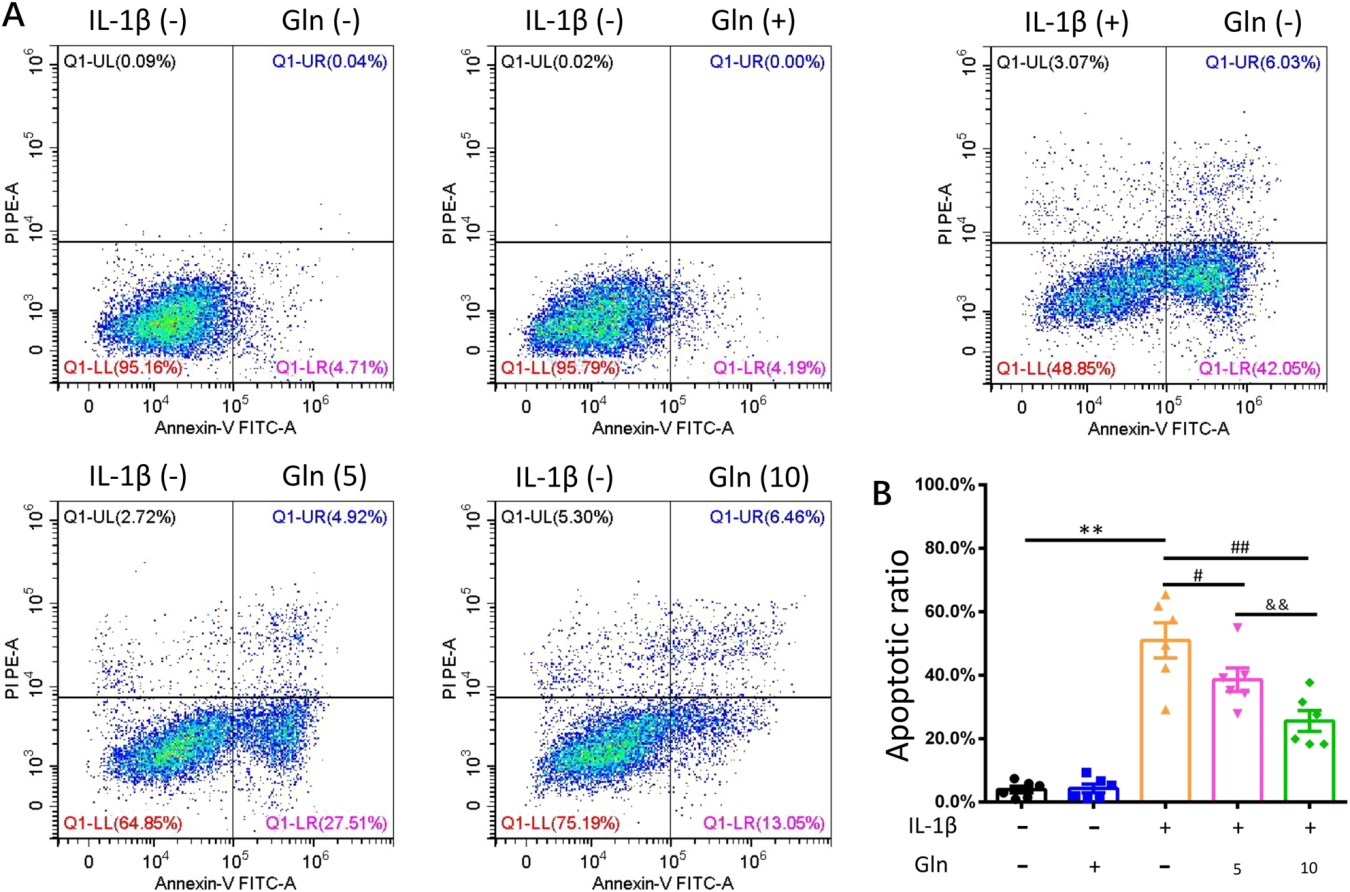
Effect of both concentrations of glutamine on the apoptotic ratio. The chondrocytes were cultured in 96-well plates and stimulated with IL-1β (10 ng/ml) for 24 h following treatment with both doses of glutamine (5, 10 μmol/l) for 2 h. (**A**) Apoptotic ratio in each group was checked using FCM. (**B**) Histogram of the apoptotic ratio of each group. All data are presented as the M ± SEM (n = 6 in each group). *p < 0.05 vs. IL-1β(−) and Gln(−) group, **p < 0.01 vs. IL-1β(−) and Gln(−) group; ^#^p < 0.05 vs. IL-1β(+) and Gln(−) group, ^##^p < 0.01 vs. IL-1β(+) and Gln(−) group; ^&^p < 0.05 vs. IL-1β(+) and Gln(5) group, ^&&^p < 0.01 vs. IL-1β(+) and Gln(5) group  by ANOVA.

To mimic the model of OA at the cellular level, we treated chondrocytes with IL-1β for 24 h. Subsequently, Gln was added to the 5 µmol/l and 10 µmol/l IL-1β + Gln experimental groups. The viability of chondrocytes was significantly decreased following IL-1β treatment. The results suggested that the model of OA at the cellular level was successful; moreover, cell viability was improved following Gln treatment for 2 h (Fig. 1E). Subsequently, we further examined the cell apoptotic ratio using FCM, and the results showed that the ratio was significantly downregulated in both Gln groups (Fig. 2A,B). These outcomes indirectly indicated that Gln protected chondrocytes against IL-1β treatment and that the effect in the high-dose group was better than that in the low-dose group.

Gln inhibits ADAMTS-5, MMP-1 and MMP-13 expression in IL-1β-treated human chondrocytes but promotes aggrecan expression.

**Figure 3:**
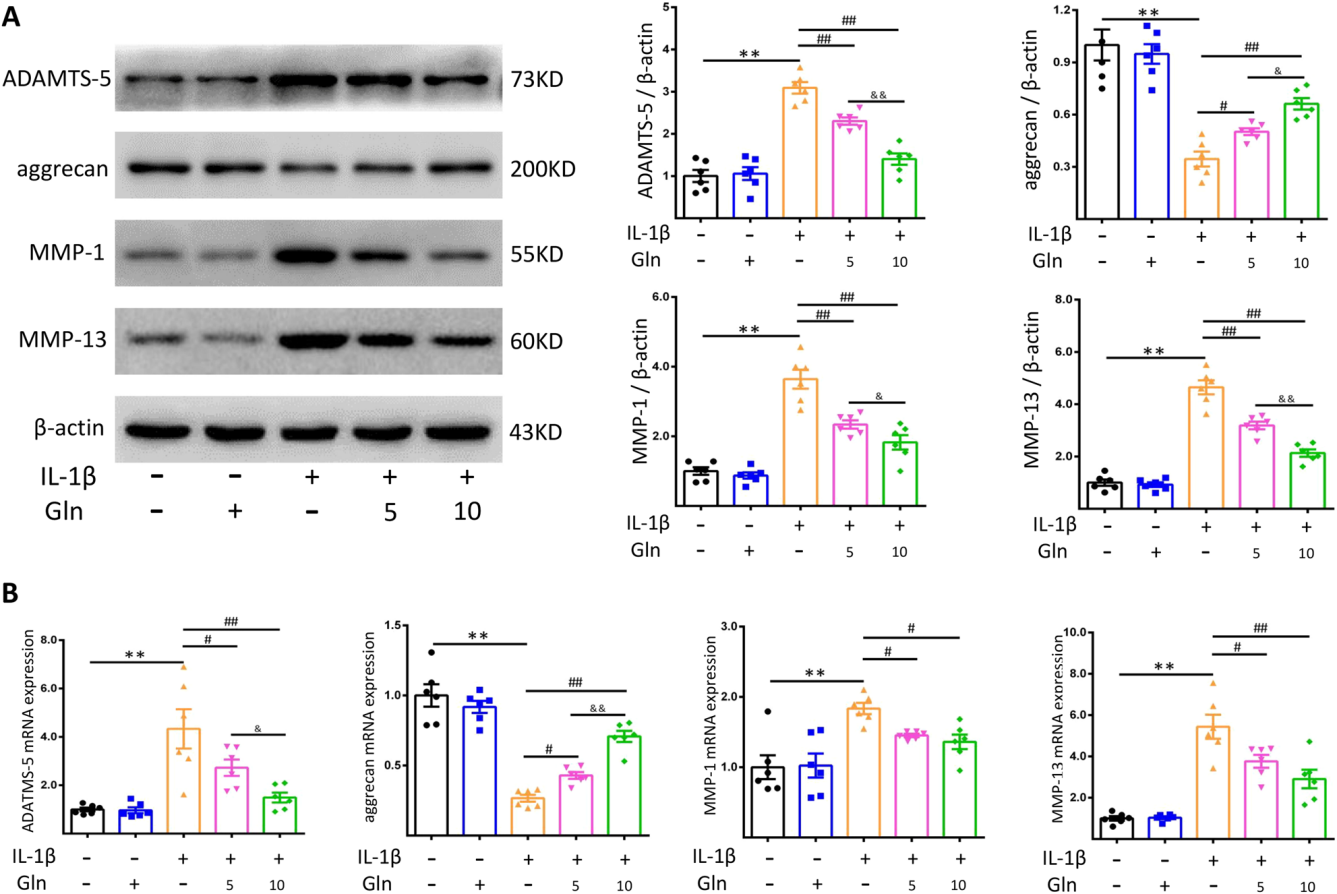
Effect of both concentrations of glutamine on ADAMTS-5, aggrecan, MMP-1, and MMP-13 in chondrocytes treated with IL-1β. Glutamine promoted the expression of a protective protein (aggrecan) and inhibited the expression of harmful proteins (ADAMTS-5, MMP-1, MMP-13). The benefit of the high concentration group was better than that of the low concentration group. (**A**) The protein expression was examined by western blot with β-actin as the internal standard. (**B**) mRNA expression levels were checked using qRT-PCR. All data are presented as the M ± SEM (n = 6 in each group). *p < 0.05 vs. IL-1β(−) and Gln(−) group, **p < 0.01 vs. IL-1β(−) and Gln(−) group; ^#^p < 0.05 vs. IL-1β(+) and Gln(−) group, ^##^p < 0.01 vs. IL-1β(+) and Gln(−) group; ^&^p < 0.05 vs. IL-1β(+) and Gln(5) group, ^&&^p < 0.01 vs. IL-1β(+) and Gln(5) group by ANOVA.

MMPs and ADAMTS-5 are the major matrix-degrading enzymes that play a pivotal role in OA pathogenesis. Aggrecan is a major structure of the cartilage extracellular matrix (ECM). Therefore, we investigated the effects of Gln on MMP-1, MMP-13, ADAMTS-5, and aggrecan mRNA and protein expression. As shown in the results, IL-1β significantly increased the expression of ADAMTS-5, MMP-1, and MMP-13 and decreased aggrecan expression in chondrocytes (Fig. 3B). However, Gln inhibited ADAMTS-5, MMP-1, and MMP-13 mRNA expression but increased aggrecan mRNA expression following IL-1β-induction of chondrocytes (Fig. 3B). To precisely measure the effects of Gln, WB was performed to examine the protein level indicated above. The results showed that Gln also promoted aggrecan expression at the protein level but downregulated ADAMTS-5, MMP-1 and MMP-13. The benefit of Gln was upregulated following IL-1β treatment and increased gradually depending on the Gln dose (Fig. 3A). These results indicated that Gln reversed the effect of IL-1β on chondrocytes.

Gln suppresses the JNK and NF-κB signaling pathways in IL-1β-treated human chondrocytes.

**Figure 4:**
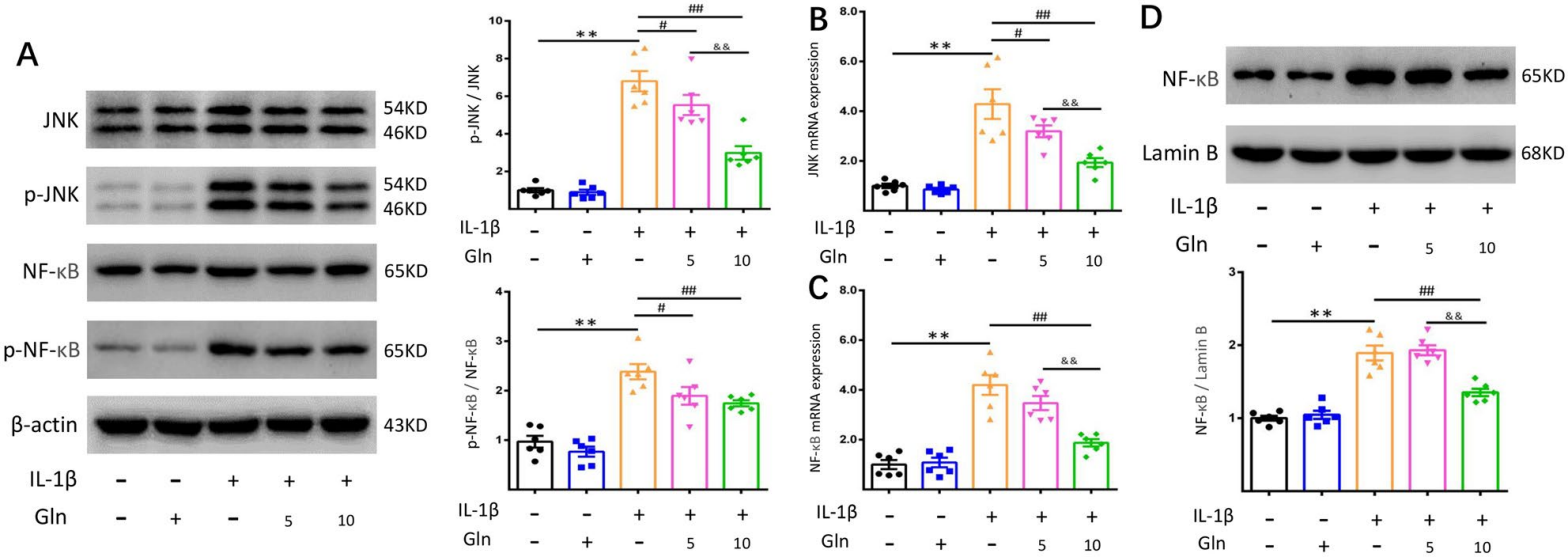
Gln suppressed IL-1β-induced JNK and NF-κB expression in chondrocytes, and the high concentration Gln exhibited a greater effect. (**A**) Protein expression levels of JNK, p-JNK, NF-κB, and p-NF-κB were examined by western blotting with β-actin as the internal standard. (**B**,**C**) JNK and NF-κB mRNA expression was measured using qRT–PCR. (**D**) The nucleoprotein expression of NF-κB was checked by western blot with Lamin B as the internal standard. All data are presented as the M ± SEM (n = 6 in each group). *p < 0.05 vs. IL-1β(−) and Gln(−) group, **p < 0.01 vs. IL-1β(−) and Gln(−) group; ^#^p < 0.05 vs. IL-1β(+) and Gln(−) group, ^##^p < 0.01 vs. IL-1β(+) and Gln(−) group; ^&^p < 0.05 vs. IL-1β(+) and Gln(5) group, ^&&^p < 0.01 vs. IL-1β(+) and Gln(5) group  by ANOVA.

To better understand the regulatory mechanisms of Gln on IL-1β-induced human chondrocytes and whether they are related to the JNK and NF-κB signaling pathways, we detected the mRNA and protein expression levels. The results showed that IL-1β markedly induced the expression of NF-κB and JNK in chondrocytes (Fig. 4B,C). Meanwhile, Gln obviously suppressed the mRNA expression of JNK and NF-κB, and there was a significant difference between both Gln groups (Fig. 4B,C). At the protein level, we measured the expression of P-JNK, JNK, P-NF-κB, and NF-κB and analysed the ratio of p-JNK/JNK and p-NF-κB/NF-κB. We found that the ratio of p-JNK/JNK was decreased after Gln treatment compared to that in the IL-1β group, and a similar result was obtained by examining p-NF-κB/NF-κB (Fig. 4A). To further explore the nuclear translocation of NF-κB, the nucleoprotein was extracted and checked. The bands showed that the ratio of NF-κB/Lamin B was significantly weakened by Gln (10 μmol/l) compared to the IL-1β group (Fig. 4D). These results further indicated that Gln attenuated JNK and NF-κB, which were induced by IL-1β.

Gln improves cartilage and enhances the OARIS score in the DMM-induced OA mouse model.

**Figure 5:**
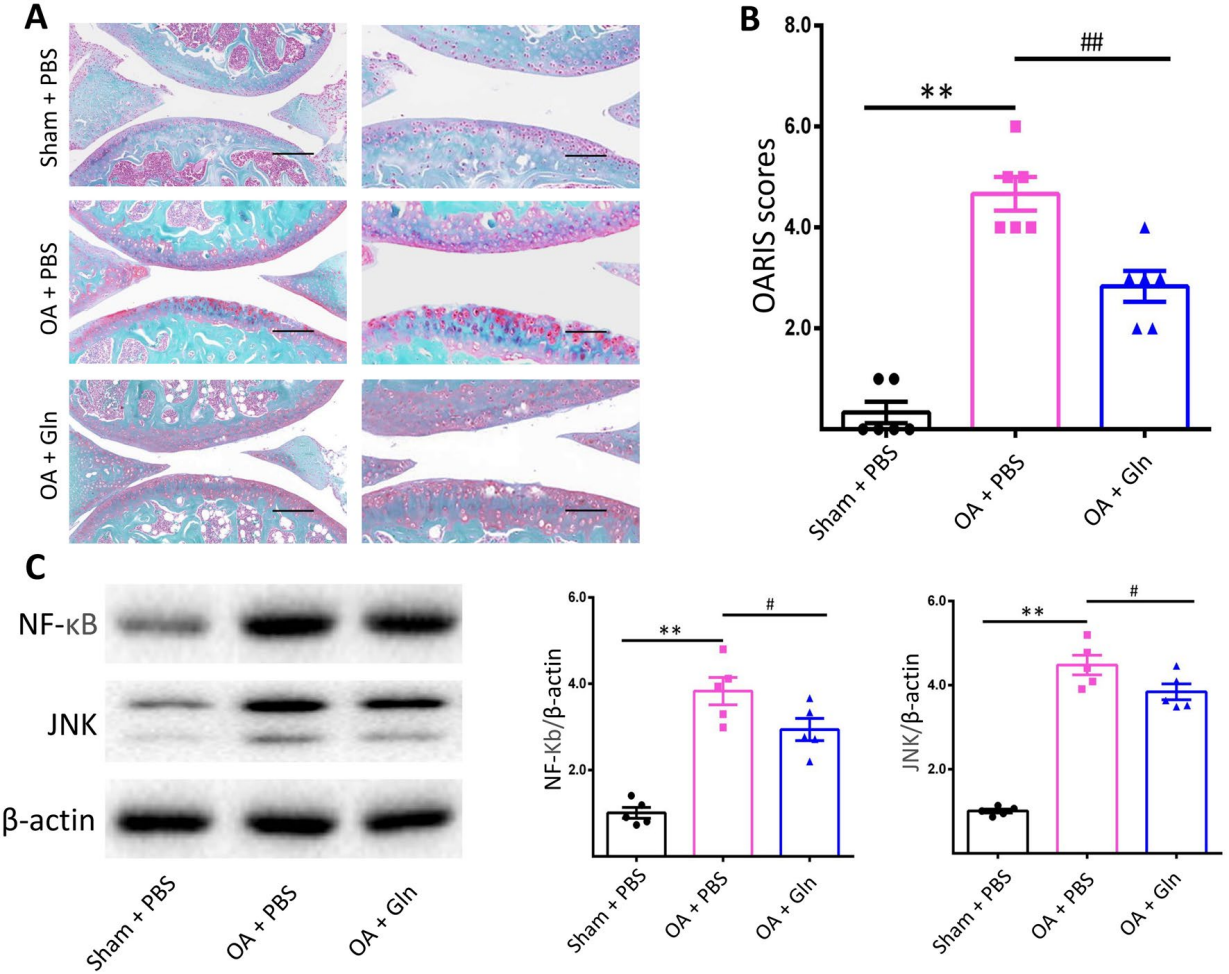
Gln inhibited the expression of JNK and NF-κB and protected articular cartilage against DMM-induced osteoarthritis. (**A**) Knee joints of mice in each group were stained with safranin-O and fast green. Scale bar, 200 μm. (**B**) OARSI scores were calculated and analysed using the Osteoarthritis Research Society International (OARSI) scoring system as follows (six OA grades): 0 = surface intact; 1 = cartilage intact; 2 = discontinuous surface; 3 = vertical fissures; 4 = erosion; 5 = denudation; and 6 = deformation. (**C**) The protein expression levels of JNK and NF-κB were examined by western blotting with β-actin as the internal standard. All data are presented as the M ± SEM (n = 5 in each group). *p < 0.05 vs. Sham + PBS group, **p < 0.01 vs. Sham + PBS group; ^#^p < 0.05 vs. OA + PBS group, ^##^p < 0.01 vs. OA + PBS group; ^&^p < 0.05 vs. IL-1β(+) and Gln(5) group, ^&&^p < 0.01 vs. IL-1β(+) and Gln(5) group by ANOVA.

To evaluate the protective effects of Gln in the progression of OA, we employed the classical animal model, in which surgical DMM was performed in C57BL/6 mice. Gln was administered by intra-articular injection once a week for 12 weeks. We used safranin-O and fast green staining to check the cartilage and measured the destruction of the cartilage layer by Osteoarthritis Research Society International. The subchondral bone is the subchondral bone plate and bone trabecular structure below the articular cartilage deposition line. The lesion of the cartilage layer was involved subchondral bone, and the result was worse than that in the sham group, however, some improvement was observed in the Gln group compared to the OA group and cartilage degeneration only occurred in the cartilage layer (Fig. 5A). Moreover, the OARSI scores showed a significant benefit between the OA group and the Gln group (Fig. 5B). Meanwhile, the protein expression levels of JNK and NF-κB were inhibited significantly with the Gln treatment (Fig. 5C). These results further suggest that Gln inhibits the JNK and NF-κB signaling pathways, improves cartilage degradation and delays the pathological process of OA.

## Discussion

OA is characterized by the progressive degeneration of articular cartilage, which is the most essential ingredient on the outermost surface of the human joint^32,33^. Chondrocytes are the only cell type in articular cartilage that are responsible for the synthesis of extracellular matrix components and maintaining the integrity of cartilage tissues^34^. The balance between cartilage synthesis and catabolism is vital for maintaining the function of articular cartilage, in which chondrocytes are encircled with plentiful aggrecan and collagen II^35,36^. Although tremendous progress has been observed in recent years for many treatments for OA, including physical activity, surgery, and drug effects, satisfactory effects against OA have not been reached^37^. Therefore, it is urgent to develop more effective and safe treatments for OA.

Glutamine is well known as a type of nonessential amino acid that is applied to treat gastric and duodenal ulcers, gastritis, etc. Accumulating reports have shown that it exerts anti-inflammation and antioxidation effects to improve and recover injured tissues^38,39^. In our study, several concentrations of Gln to treat chondrocytes for 24 h showed that no significant cytotoxicity was observed under 5 and 10 µmol/l Gln. Next, the effect of Gln on OA must be investigated in vitro.

Previous studies have demonstrated that IL-1β enhances the expression of metalloproteinases, such as MMP1, MMP13, and ADAMTS-5, in chondrocytes^40^. Moreover, IL-1β-induced chondrocytes increase ADAMTS-5 expression and reduce proteoglycan production^41^. Hence, we used both doses of Gln to treat the OA chondrocyte model, which was induced with IL-1β. The results showed that the cell viability in both Gln groups was significantly improved, which verified that Gln can benefit OA chondrocytes.

Accumulating studies have demonstrated that the pathological process of OA is the imbalance of cartilage synthesis and catabolis accompanying with enhancement of ADAMTS-5, MMP-1, and MMP-13, which can almost completely degrade various protein components in the extracellular matrix (ECM), including proteoglycans and collagen II^42–45^. Therefore, inhibiting ADAMTS-5 and MMPs and promoting proteoglycans may be a potential treatment strategy for OA. Our experimental results showed that destructive factors (MMP-1, MMP-13, and ADAMTS-5) were expressed at low levels in both Gln groups at the mRNA and protein levels and a protective factor (aggrecan) was highly expressed; however, significant differences were observed between the groups. These outcomes indicated that Gln protected chondrocytes against OA by promoting aggrecan and inhibiting MMP-1, MMP-13 and ADAMTS-5 depending on the doses of Gln.

JNK and NF-κB are classical signaling pathways involved in OA progression that regulate the expression of the above factors^46,47^. NF-κB activation determines the expression of a wide range of catabolic mediators in articular cartilage. Among the downstream signaling pathways triggered by inflammatory factors, the NF-κB signaling pathway is the central regulator of catabolic actions, mediating crucial events in the inflammatory responses of chondrocytes and leading to extracellular matrix (ECM) damage and cartilage erosion^34^. Scientific studies have shown that JNK is activated (phosphorylated) in OA and plays an essential role in cartilage destruction. Activation of JNK causes the phosphorylation of c-Jun, which causes decreased proteoglycan synthesis and enhanced production of matrix metalloproteinase 13 (MMP-13)^46^. Our data showed that the mRNA and protein expression levels of JNK and NF-κB were downregulated following Gln treatment. Moreover, the ratio of NF-κB/Lamin B was reduced. In summary, these results further indicated that Gln protected the chondrocytes against IL-1β treatment through the JNK and NF-κB signaling pathways.

We further explored the effect of Gln in a mouse OA model induced by classical DMM surgery, in which the number of IL-1β-positive chondrocytes was greatly increased^48^. We used 100 mM Gln for intra-articular injection according to the other experiments and our conditions^31,49^. Safranin O and Fast Green can stain cartilage and subchondral bone red or green, respectively, to evaluate the overall staging of OA progression combined with the OARSI cartilage assessment system^50^. The slides of the mouse joints showed that cartilage degradation was less severe in the Gln group than that in the OA group and the OARSI scores improved greatly. Combining the upregulation of JNK and NF-κb in vivo, the results suggested that Gln could significantly attenuate the process of OA through the JNK and NF-κb signaling pathways.

## Conclusion

Our outcomes are the first to demonstrate that Gln benefits OA by inhibiting the JNK and NF-κB signaling pathways. This finding on the protective effects of Gln may support further investigations on potential therapeutic interventions for OA.

## Supplementary Information

Supplementary Information 1.

Supplementary Information 2.

Supplementary Information 3.

## Data Availability

All data used or analysed during the current study are available from the corresponding author on reasonable request.

## References

[1] Li J, Zhang B, Liu WX (2020). Metformin limits osteoarthritis development and progression through activation of AMPK signaling. Ann. Rheum. Dis..

[2] Glyn-Jones S, Palmer AJ, Agricola R (2015). Osteoarthritis. Lancet.

[3] Karsdal MA, Michaelis M, Ladel C (2016). Disease-modifying treatments for osteoarthritis (DMOADs) of the knee and hip: Lessons learned from failures and opportunities for the future. Osteoarthr. Cartil..

[4] Vos T, Flaxman AD, Naghavi M (2012). Years lived with disability (YLDs) for 1160 sequelae of 289 diseases and injuries 1990–2010: A systematic analysis for the Global Burden of Disease Study 2010. Lancet.

[5] Huang W, Cheng C, Shan WS (2020). Knockdown of SGK1 alleviates the IL-1β-induced chondrocyte anabolic and catabolic imbalance by activating FoxO1-mediated autophagy in human chondrocytes. FEBS J..

[6] Robinson WH, Lepus CM, Wang Q (2016). Low-grade inflammation as a key mediator of the pathogenesis of osteoarthritis. Nat. Rev. Rheumatol..

[7] Zhou PH, Qiu B, Deng RH, Li HJ, Xu XF, Shang XF (2018). Chondroprotective effects of hyaluronic acid-chitosan nanoparticles containing plasmid DNA encoding cytokine response modifier A in a rat knee osteoarthritis model. Cell Physiol. Biochem..

[8] Abramson SB, Attur M, Amin AR, Clancy R (2001). Nitric oxide and inflammatory mediators in the perpetuation of osteoarthritis. Curr. Rheumatol. Rep..

[9] Jin T, Wu D, Liu XM (2020). Intra-articular delivery of celastrol by hollow mesoporous silica nanoparticles for pH-sensitive anti-inflammatory therapy against knee osteoarthritis. J. Nanobiotechnol..

[10] Zhou S, Shi J, Wen H, Xie W, Han X, Li H (2020). A chondroprotective effect of moracin on IL-1β-induced primary rat chondrocytes and an osteoarthritis rat model through Nrf2/HO-1 and NF-κB axes. Food Funct..

[11] Jotanovic Z, Mihelic R, Sestan B, Dembic Z (2012). Role of interleukin-1 inhibitors in osteoarthritis: An evidence-based review. Drugs Aging..

[12] Hall JC, Heel K, McCauley R (1996). Glutamine. Br. J. Surg..

[13] de Oliveira GP, Kitoko JZ, de Souza Lima-Gomes P (2019). Glutamine therapy reduces inflammation and extracellular trap release in experimental acute respiratory distress syndrome of pulmonary origin. Nutrients.

[14] Li Y, Wu XB, Li JG (2014). Enteral supplementation of alanyl-glutamine attenuates the up-regulation of beta-defensin-2 protein in lung injury induced by intestinal ischemia reperfusion in rats. Int. J. Surg..

[15] Shah AM, Wang Z, Ma J (2020). Glutamine metabolism and its role in immunity, a comprehensive review. Animals (Basel)..

[16] Cruzat V, Macedo Rogero M, Noel Keane K, Curi R, Newsholme P (2018). Glutamine: Metabolism and immune function, supplementation and clinical translation. Nutrients.

[17] Huang H, Lin Z, Zeng Y, Lin X, Zhang Y (2019). Probiotic and glutamine treatments attenuate alcoholic liver disease in a rat model. Exp. Ther. Med..

[18] Gong ZY, Yuan ZQ, Dong ZW, Peng YZ (2017). Glutamine with probiotics attenuates intestinal inflammation and oxidative stress in a rat burn injury model through altered iNOS gene aberrant methylation. Am. J. Transl. Res..

[19] Wang J, Zhou J, Bai S (2020). Combination of glutamine and ulinastatin treatments greatly improves sepsis outcomes. J. Inflamm. Res..

[20] Crespo I, San-Miguel B, Prause C (2012). Glutamine treatment attenuates endoplasmic reticulum stress and apoptosis in TNBS-induced colitis. PLoS ONE.

[21] Ulivi V, Giannoni P, Gentili C, Cancedda R, Descalzi F (2008). p38/NF-kB-dependent expression of COX-2 during differentiation and inflammatory response of chondrocytes. J. Cell Biochem..

[22] de Andrés MC, Takahashi A, Oreffo RO (2016). Demethylation of an NF-κB enhancer element orchestrates iNOS induction in osteoarthritis and is associated with altered chondrocyte cell cycle. Osteoarthr. Cartil..

[23] Hu ZC, Xie ZJ, Tang Q (2018). Hydroxysafflor yellow A (HSYA) targets the NF-κB and MAPK pathways and ameliorates the development of osteoarthritis. Food Funct..

[24] Liacini A, Sylvester J, Li WQ, Zafarullah M (2002). Inhibition of interleukin-1-stimulated MAP kinases, activating protein-1 (AP-1) and nuclear factor kappa B (NF-kappa B) transcription factors down-regulates matrix metalloproteinase gene expression in articular chondrocytes. Matrix Biol..

[25] Yaykasli KO, Hatipoglu OF, Yaykasli E (2015). Leptin induces ADAMTS-4, ADAMTS-5, and ADAMTS-9 genes expression by mitogen-activated protein kinases and NF-ĸB signaling pathways in human chondrocytes. Cell Biol. Int..

[26] Yang P, Tan J, Yuan Z, Meng G, Bi L, Liu J (2016). Expression profile of cytokines and chemokines in osteoarthritis patients: Proinflammatory roles for CXCL8 and CXCL11 to chondrocytes. Int. Immunopharmacol..

[27] Loeser RF, Chubinskaya S, Pacione C, Im HJ (2005). Basic fibroblast growth factor inhibits the anabolic activity of insulin-like growth factor 1 and osteogenic protein 1 in adult human articular chondrocytes. Arthritis Rheum..

[28] Johnson GL, Nakamura K (2007). The c-jun kinase/stress-activated pathway: Regulation, function and role in human disease. Biochim. Biophys. Acta..

[29] Latourte A, Cherifi C, Maillet J (2017). Systemic inhibition of IL-6/Stat3 signaling protects against experimental osteoarthritis. Ann. Rheum. Dis..

[30] Jahangir S, Eglin D, Pötter N (2020). Inhibition of hypertrophy and improving chondrocyte differentiation by MMP-13 inhibitor small molecule encapsulated in alginate-chondroitin sulfate-platelet lysate hydrogel. Stem Cell Res. Ther..

[31] Fujita S, Arai Y, Nakagawa S (2012). Combined microwave irradiation and intraarticular glutamine administration-induced HSP70 expression therapy prevents cartilage degradation in a rat osteoarthritis model. J. Orthop. Res..

[32] Akkiraju H, Nohe A (2015). Role of chondrocytes in cartilage formation, progression of osteoarthritis and cartilage regeneration. J. Dev. Biol..

[33] Neuhold LA, Killar L, Zhao W (2001). Postnatal expression in hyaline cartilage of constitutively active human collagenase-3 (MMP-13) induces osteoarthritis in mice. J. Clin. Investig..

[34] Lu W, Ding Z, Liu F (2019). Dopamine delays articular cartilage degradation in osteoarthritis by negative regulation of the NF-κB and JAK2/STAT3 signaling pathways. Biomed. Pharmacother..

[35] Musumeci G, Loreto C, Imbesi R (2014). Advantages of exercise in rehabilitation, treatment and prevention of altered morphological features in knee osteoarthritis. A narrative review. Histol. Histopathol..

[36] Ding Z, Lu W, Dai C (2020). The CRD of Frizzled 7 exhibits chondroprotective effects in osteoarthritis via inhibition of the canonical Wnt3a/β-catenin signaling pathway. Int. Immunopharmacol..

[37] Szychlinska MA, Castrogiovanni P, Trovato FM (2019). Physical activity and Mediterranean diet based on olive tree phenolic compounds from two different geographical areas have protective effects on early osteoarthritis, muscle atrophy and hepatic steatosis. Eur. J. Nutr..

[38] El-Lekawy AM, Abdallah DM, El-Abhar HS (2019). Alanyl-glutamine heals indomethacin-induced gastric ulceration in rats via antisecretory and anti-apoptotic mechanisms. J. Pediatr. Gastroenterol. Nutr..

[39] Takagi K, Takeuchi K, Nakamura K, Morita A, Okabe S (1974). Effects of an antiulcer agent *N*-acetyl-l-glutamine aluminum complex (KW-110) on the duodenal and gastric ulcer models in the rat. Jpn. J. Pharmacol..

[40] Zheng W, Tao Z, Chen C (2017). Plumbagin prevents IL-1β-induced inflammatory response in human osteoarthritis chondrocytes and prevents the progression of osteoarthritis in mice. Inflammation.

[41] Alberton P, Dugonitsch HC, Hartmann B (2019). Aggrecan hypomorphism compromises articular cartilage biomechanical properties and is associated with increased incidence of spontaneous osteoarthritis. Int. J. Mol. Sci..

[42] Song RH, Tortorella MD, Malfait AM (2007). Aggrecan degradation in human articular cartilage explants is mediated by both ADAMTS-4 and ADAMTS-5. Arthritis Rheum..

[43] Glasson SS, Askew R, Sheppard B (2005). Deletion of active ADAMTS5 prevents cartilage degradation in a murine model of osteoarthritis. Nature.

[44] Burrage PS, Mix KS, Brinckerhoff CE (2006). Matrix metalloproteinases: Role in arthritis. Front. Biosci..

[45] Zeng GQ, Chen AB, Li W, Song JH, Gao CY (2015). High MMP-1, MMP-2, and MMP-9 protein levels in osteoarthritis. Genet. Mol. Res..

[46] Ge HX, Zou FM, Li Y, Liu AM, Tu M (2017). JNK pathway in osteoarthritis: Pathological and therapeutic aspects. J. Recept. Signal Transduct. Res..

[47] Qiu B, Xu X, Yi P, Hao Y (2020). Curcumin reinforces MSC-derived exosomes in attenuating osteoarthritis via modulating the miR-124/NF-kB and miR-143/ROCK1/TLR9 signaling pathways. J. Cell Mol. Med..

[48] Dai J, Yu D, Wang Y (2017). Kdm6b regulates cartilage development and homeostasis through anabolic metabolism. Ann. Rheum. Dis..

[49] Tonomura H, Takahashi KA, Mazda O (2006). Glutamine protects articular chondrocytes from heat stress and NO-induced apoptosis with HSP70 expression. Osteoarthr. Cartil..

[50] Pritzker KP, Gay S, Jimenez SA (2006). Osteoarthritis cartilage histopathology: Grading and staging. Osteoarthr. Cartil..

