# Supplementary material for: Glutamine exerts a protective effect on osteoarthritis development by inhibiting the Jun N-terminal kinase and nuclear factor kappa-B signaling pathways
Source: Sci Rep. 2022 Jul 13;12:11957. doi: 10.1038/s41598-022-16093-7 (PMC9279466; doi:10.1038/s41598-022-16093-7)
Supplement: Supplementary file 1 — Supplementary Information 1. [file 41598_2022_16093_MOESM1_ESM.pdf]

cell viability

IL-1β	无	无	有	有	有
Gln	无	有	无	有	有有
	2.78	2.85	1.47	1.52	1.97
	2.72	2.74	1.86	2.22	2.43
时间	2.89	2.99	1.55	1.76	2.00
	2.88	2.91	1.77	1.99	2.50
	2.97	2.77	1.47	2.36	2.34
	2.75	2.98	1.43	1.53	1.88

FCM

IL-1β	无	无	有	有	有
Gln	无	有	无	有	有有
时间	95.16	95.79	48.48	64.85	75.19
	98.13	93.29	57.38	57.76	81.67
	94.83	97.96	40.16	68.12	72.41
	95.16	95.79	48.48	64.85	75.19
	98.13	93.29	57.38	57.76	81.67
	94.83	97.96	40.16	68.12	72.41

WB

IL-1β	无	无	有	有	有
Gln	无	有	无	有	有有
JNK	1451	1458	1965	1779	1556
	1005	1375	1862	1983	1305
	1728	1781	1639	1524	1344
	1343	1576	1718	1842	1811
	1470	1882	1765	1898	1735
	1493	1504	1677	1664	1644
actin	912	819	814	775	851
p-JNK	228	264	1773	1346	647
	243	311	1621	1478	986
	234	157	2166	1932	501
	208	202	1699	1497	790
	158	174	2401	1389	698
	230	203	1738	1601	760
NF-KB	1074	942	1207	1004	1151
	979	1064	1065	967	909
	1062	1183	1074	881	985
	1035	1159	1113	1210	845
	908	956	960	1047	1280
	1143	984	1023	1123	1045
p-NF-KB	353	303	1038	781	770
	527	429	863	499	716
	445	219	1352	934	665
	244	227	1121	1028	634
	476	405	920	730	821
	389	357	986	851	806
ADATMS-5	417	524	1275	1075	824
	293	418	1509	1172	478
	697	722	1121	977	607
	325	225	1178	1078	973
	663	689	1387	921	790

	542	547	1348	964	776
actin	549	553	474	503	595
aggrecan	610	505	264	312	493
	876	762	306	350	417
	829	737	165	337	433
	513	647	183	293	464
	559	583	127	318	566
	697	669	227	267	543
MMP-1	393	356	1345	719	404
	255	205	1031	871	708
	476	327	1550	667	846
	263	290	937	870	624
	477	456	1193	756	960
	383	337	1356	919	896
MMP-13	317	321	1463	1014	805
	232	440	1153	1200	980
	400	405	1601	1071	1008
	536	227	1750	867	646
	423	290	1337	1170	774
	304	375	1549	1123	886
核NF-KB	855	807	1585	1562	1058
	941	701	1833	1401	908
	970	1010	1771	1342	1135
	777	762	1309	1693	892
	898	890	1408	1465	987
	843	803	1459	1418	965
Lamin b	1598	1440	1449	1394	1333

	Sham + PBS	OA+ PBS	OA+Gln
NF-KB	588	2636	1990
	910	3210	1620
	469	1998	2184
	521	2753	1651
	768	2207	1307
actin	2801	2907	2588

	Sham + PBS	OA+ PBS	OA+Gln
JNK	930	4582	3502
	993	3754	3806
	806	4230	2978
	1058	3923	3007
	879	4991	3108

PCR

A7/A1		NFKB	GAPDH	28.75	18.95
A8/A2		NFKB	GAPDH	27.57	18.92
A9/A3		NFKB	GAPDH	28.39	18.68
A10/A4		NFKB	GAPDH	27.22	18.68
A11/A5		NFKB	GAPDH	27.17	18.80
A12/A6		NFKB	GAPDH	28.64	18.98
B7/B1	Gln	NFKB	GAPDH	27.09	18.71
B8/B2	Gln	NFKB	GAPDH	28.54	18.53

B9/B3	Gln	NFKB	GAPDH	27.36	18.81
B10/B4	Gln	NFKB	GAPDH	27.61	18.25
B11/B5	Gln	NFKB	GAPDH	26.83	18.51
B12/B6	Gln	NFKB	GAPDH	26.95	17.51
C7/C1	IL-1β	NFKB	GAPDH	25.70	19.17
C8/C2	IL-1β	NFKB	GAPDH	25.53	18.74
C9/C3	IL-1β	NFKB	GAPDH	25.53	18.03
C10/C4	IL-1β	NFKB	GAPDH	25.61	18.84
C11/C5	IL-1β	NFKB	GAPDH	26.28	19.03
C12/C6	IL-1β	NFKB	GAPDH	26.31	19.38
D7/D1	IL-1β+Gln(	NFKB	GAPDH	26.31	19.33
D8/D2	IL-1β+Gln(	NFKB	GAPDH	27.19	19.78
D9/D3	IL-1β+Gln(	NFKB	GAPDH	26.38	18.85
D10/D4	IL-1β+Gln(	NFKB	GAPDH	25.04	17.53
D11/D5	IL-1β+Gln(	NFKB	GAPDH	26.63	19.75
D12/D6	IL-1β+Gln(	NFKB	GAPDH	26.58	19.54
E7/E1	IL-1β+Gln(	NFKB	GAPDH	26.25	18.12
E8/E2	IL-1β+Gln(	NFKB	GAPDH	26.64	18.67
E9/E3	IL-1β+Gln(	NFKB	GAPDH	27.47	18.86
E10/E4	IL-1β+Gln(	NFKB	GAPDH	27.52	19.68
E11/E5	IL-1β+Gln(	NFKB	GAPDH	26.82	18.97
E12/E6	IL-1β+Gln(	NFKB	GAPDH	27.31	19.02
A7/A1		JNK	GAPDH	36.93	23.56
A8/A2		JNK	GAPDH	36.69	23.08
A9/A3		JNK	GAPDH	36.63	23.11
A10/A4		JNK	GAPDH	36.92	23.05
A11/A5		JNK	GAPDH	37.18	23.13
A12/A6		JNK	GAPDH	36.64	23.44
B7/B1	Gln	JNK	GAPDH	37.53	23.37
B8/B2	Gln	JNK	GAPDH	37.18	23.19
B9/B3	Gln	JNK	GAPDH	36.34	22.73
B10/B4	Gln	JNK	GAPDH	37.29	23.29
B11/B5	Gln	JNK	GAPDH	37.57	23.91
B12/B6	Gln	JNK	GAPDH	37.20	23.72
C7/C1	IL-1β	JNK	GAPDH	34.99	23.97
C8/C2	IL-1β	JNK	GAPDH	35.54	23.62
C9/C3	IL-1β	JNK	GAPDH	35.27	23.20
C10/C4	IL-1β	JNK	GAPDH	34.24	23.29
C11/C5	IL-1β	JNK	GAPDH	35.27	23.37
C12/C6	IL-1β	JNK	GAPDH	35.30	23.89
D7/D1	IL-1β+Gln(	JNK	GAPDH	35.79	23.76
D8/D2	IL-1β+Gln(	JNK	GAPDH	35.24	23.56
D9/D3	IL-1β+Gln(	JNK	GAPDH	35.59	23.63
D10/D4	IL-1β+Gln(	JNK	GAPDH	35.22	23.51
D11/D5	IL-1β+Gln(	JNK	GAPDH	35.63	23.20
D12/D6	IL-1β+Gln(	JNK	GAPDH	35.21	23.48
E7/E1	IL-1β+Gln(	JNK	GAPDH	35.79	23.17
E8/E2	IL-1β+Gln(	JNK	GAPDH	35.91	23.29
E9/E3	IL-1β+Gln(	JNK	GAPDH	36.79	23.51
E10/E4	IL-1β+Gln(	JNK	GAPDH	35.27	22.49
E11/E5	IL-1β+Gln(	JNK	GAPDH	36.54	24.13
E12/E6	IL-1β+Gln(	JNK	GAPDH	36.09	23.85
A7/A1		ADAMTS-5	GAPDH	31.84	22.17
A8/A2		ADAMTS-5	GAPDH	31.80	21.85
A9/A3		ADAMTS-5	GAPDH	31.84	21.82

A10/A4		ADAMTS-5	GAPDH	31.23	21.81
A11/A5		ADAMTS-5	GAPDH	31.33	21.77
A12/A6		ADAMTS-5	GAPDH	31.46	21.34
B7/B1	Gln	ADAMTS-5	GAPDH	32.15	22.92
B8/B2	Gln	ADAMTS-5	GAPDH	31.50	22.02
B9/B3	Gln	ADAMTS-5	GAPDH	32.03	21.97
B10/B4	Gln	ADAMTS-5	GAPDH	31.94	21.95
B11/B5	Gln	ADAMTS-5	GAPDH	32.72	22.45
B12/B6	Gln	ADAMTS-5	GAPDH	32.54	22.36
C7/C1	IL-1β	ADAMTS-5	GAPDH	31.66	22.59
C8/C2	IL-1β	ADAMTS-5	GAPDH	30.09	22.61
C9/C3	IL-1β	ADAMTS-5	GAPDH	29.72	21.71
C10/C4	IL-1β	ADAMTS-5	GAPDH	29.93	22.95
C11/C5	IL-1β	ADAMTS-5	GAPDH	30.70	22.57
C12/C6	IL-1β	ADAMTS-5	GAPDH	30.19	23.03
D7/D1	IL-1β+Gln(	ADAMTS-5	GAPDH	31.28	22.81
D8/D2	IL-1β+Gln(	ADAMTS-5	GAPDH	31.56	22.68
D9/D3	IL-1β+Gln(	ADAMTS-5	GAPDH	31.25	23.33
D10/D4	IL-1β+Gln(	ADAMTS-5	GAPDH	31.58	22.62
D11/D5	IL-1β+Gln(	ADAMTS-5	GAPDH	31.96	24.03
D12/D6	IL-1β+Gln(	ADAMTS-5	GAPDH	31.61	23.49
E7/E1	IL-1β+Gln(	ADAMTS-5	GAPDH	32.87	23.12
E8/E2	IL-1β+Gln(	ADAMTS-5	GAPDH	33.07	23.17
E9/E3	IL-1β+Gln(	ADAMTS-5	GAPDH	32.49	23.79
E10/E4	IL-1β+Gln(	ADAMTS-5	GAPDH	32.29	23.14
E11/E5	IL-1β+Gln(	ADAMTS-5	GAPDH	32.01	23.28
E12/E6	IL-1β+Gln(	ADAMTS-5	GAPDH	32.78	23.45

A7/A1		aggrecan	GAPDH	36.25	23.75
A8/A2		aggrecan	GAPDH	35.73	23.37
A9/A3		aggrecan	GAPDH	35.47	23.02
A10/A4		aggrecan	GAPDH	35.28	23.18
A11/A5		aggrecan	GAPDH	35.91	23.09
A12/A6		aggrecan	GAPDH	36.47	23.64
B7/B1	Gln	aggrecan	GAPDH	35.81	23.23
B8/B2	Gln	aggrecan	GAPDH	35.93	23.41
B9/B3	Gln	aggrecan	GAPDH	35.09	22.19
B10/B4	Gln	aggrecan	GAPDH	35.85	23.42
B11/B5	Gln	aggrecan	GAPDH	35.87	23.13
B12/B6	Gln	aggrecan	GAPDH	35.43	22.89
C7/C1	IL-1β	aggrecan	GAPDH	38.08	23.94
C8/C2	IL-1β	aggrecan	GAPDH	38.00	23.91
C9/C3	IL-1β	aggrecan	GAPDH	38.48	23.76
C10/C4	IL-1β	aggrecan	GAPDH	37.73	23.59
C11/C5	IL-1β	aggrecan	GAPDH	38.11	23.28
C12/C6	IL-1β	aggrecan	GAPDH	37.69	23.05
D7/D1	IL-1β+Gln(	aggrecan	GAPDH	37.51	23.46
D8/D2	IL-1β+Gln(	aggrecan	GAPDH	37.13	23.64
D9/D3	IL-1β+Gln(	aggrecan	GAPDH	37.45	23.78
D10/D4	IL-1β+Gln(	aggrecan	GAPDH	37.17	23.64
D11/D5	IL-1β+Gln(	aggrecan	GAPDH	37.22	23.46
D12/D6	IL-1β+Gln(	aggrecan	GAPDH	36.88	23.07
E7/E1	IL-1β+Gln(	aggrecan	GAPDH	36.59	23.63
E8/E2	IL-1β+Gln(	aggrecan	GAPDH	36.34	23.34
E9/E3	IL-1β+Gln(	aggrecan	GAPDH	36.48	23.68
E10/E4	IL-1β+Gln(	aggrecan	GAPDH	36.14	22.74
E11/E5	IL-1β+Gln(	aggrecan	GAPDH	36.97	24.14

E12/E6	IL-1β+Gln(	aggrecan	GAPDH	37.45	24.45
A7/A1		MMP1	GAPDH	38.54	21.55
A8/A2		MMP1	GAPDH	39.10	21.88
A9/A3		MMP1	GAPDH	39.63	22.02
A10/A4		MMP1	GAPDH	39.74	22.11
A11/A5		MMP1	GAPDH	39.18	21.77
A12/A6		MMP1	GAPDH	38.76	22.50
B7/B1	Gln	MMP1	GAPDH	39.71	21.79
B8/B2	Gln	MMP1	GAPDH	39.07	22.02
B9/B3	Gln	MMP1	GAPDH	39.19	21.97
B10/B4	Gln	MMP1	GAPDH	39.81	21.95
B11/B5	Gln	MMP1	GAPDH	39.14	22.65
B12/B6	Gln	MMP1	GAPDH	39.87	23.35
C7/C1	IL-1β	MMP1	GAPDH	38.87	22.69
C8/C2	IL-1β	MMP1	GAPDH	39.33	23.21
C9/C3	IL-1β	MMP1	GAPDH	39.58	23.11
C10/C4	IL-1β	MMP1	GAPDH	39.27	22.95
C11/C5	IL-1β	MMP1	GAPDH	39.55	23.27
C12/C6	IL-1β	MMP1	GAPDH	39.81	23.78
D7/D1	IL-1β+Gln(	MMP1	GAPDH	39.93	23.42
D8/D2	IL-1β+Gln(	MMP1	GAPDH	39.94	23.37
D9/D3	IL-1β+Gln(	MMP1	GAPDH	39.99	23.34
D10/D4	IL-1β+Gln(	MMP1	GAPDH	39.95	23.44
D11/D5	IL-1β+Gln(	MMP1	GAPDH	39.79	23.18
D12/D6	IL-1β+Gln(	MMP1	GAPDH	39.13	22.59
E7/E1	IL-1β+Gln(	MMP1	GAPDH	38.72	22.11
E8/E2	IL-1β+Gln(	MMP1	GAPDH	39.23	22.88
E9/E3	IL-1β+Gln(	MMP1	GAPDH	38.50	21.33
E10/E4	IL-1β+Gln(	MMP1	GAPDH	39.01	22.52
E11/E5	IL-1β+Gln(	MMP1	GAPDH	39.04	22.23
E12/E6	IL-1β+Gln(	MMP1	GAPDH	39.47	22.82
A7/A1		MMP13	GAPDH	37.02	23.74
A8/A2		MMP13	GAPDH	36.07	23.54
A9/A3		MMP13	GAPDH	36.38	23.76
A10/A4		MMP13	GAPDH	35.70	23.59
A11/A5		MMP13	GAPDH	35.61	23.28
A12/A6		MMP13	GAPDH	36.54	23.79
B7/B1	Gln	MMP13	GAPDH	35.70	23.14
B8/B2	Gln	MMP13	GAPDH	36.01	23.51
B9/B3	Gln	MMP13	GAPDH	34.93	22.59
B10/B4	Gln	MMP13	GAPDH	36.30	23.32
B11/B5	Gln	MMP13	GAPDH	35.50	23.03
B12/B6	Gln	MMP13	GAPDH	35.87	23.56
C7/C1	IL-1β	MMP13	GAPDH	33.27	23.63
C8/C2	IL-1β	MMP13	GAPDH	33.21	23.34
C9/C3	IL-1β	MMP13	GAPDH	33.81	23.68
C10/C4	IL-1β	MMP13	GAPDH	33.52	22.74
C11/C5	IL-1β	MMP13	GAPDH	34.56	24.24
C12/C6	IL-1β	MMP13	GAPDH	33.81	23.59
D7/D1	IL-1β+Gln(	MMP13	GAPDH	34.28	23.66
D8/D2	IL-1β+Gln(	MMP13	GAPDH	34.31	23.44
D9/D3	IL-1β+Gln(	MMP13	GAPDH	34.23	23.78
D10/D4	IL-1β+Gln(	MMP13	GAPDH	34.98	23.74
D11/D5	IL-1β+Gln(	MMP13	GAPDH	33.90	23.46
D12/D6	IL-1β+Gln(	MMP13	GAPDH	34.21	23.78

E7/E1	IL-1β+Gln(	MMP13	GAPDH	35.17	24.08
E8/E2	IL-1β+Gln(	MMP13	GAPDH	34.86	23.03
E9/E3	IL-1β+Gln(	MMP13	GAPDH	34.69	24.37
E10/E4	IL-1β+Gln(	MMP13	GAPDH	35.40	23.79
E11/E5	IL-1β+Gln(	MMP13	GAPDH	35.18	24.31
E12/E6	IL-1β+Gln(	MMP13	GAPDH	34.65	23.74

OARSI评分

normal	OA	Gln
0.00	6.00	3.00
1.00	5.00	2.00
0.00	4.00	4.00
0.00	4.00	3.00
1.00	5.00	3.00
0.00	4.00	2.00
