# Supplementary figures and images for: Glutamine exerts a protective effect on osteoarthritis development by inhibiting the Jun N-terminal kinase and nuclear factor kappa-B signaling pathways
Source: Sci Rep. 2022 Jul 13;12:11957. doi: 10.1038/s41598-022-16093-7 (PMC9279466; doi:10.1038/s41598-022-16093-7)

Actin

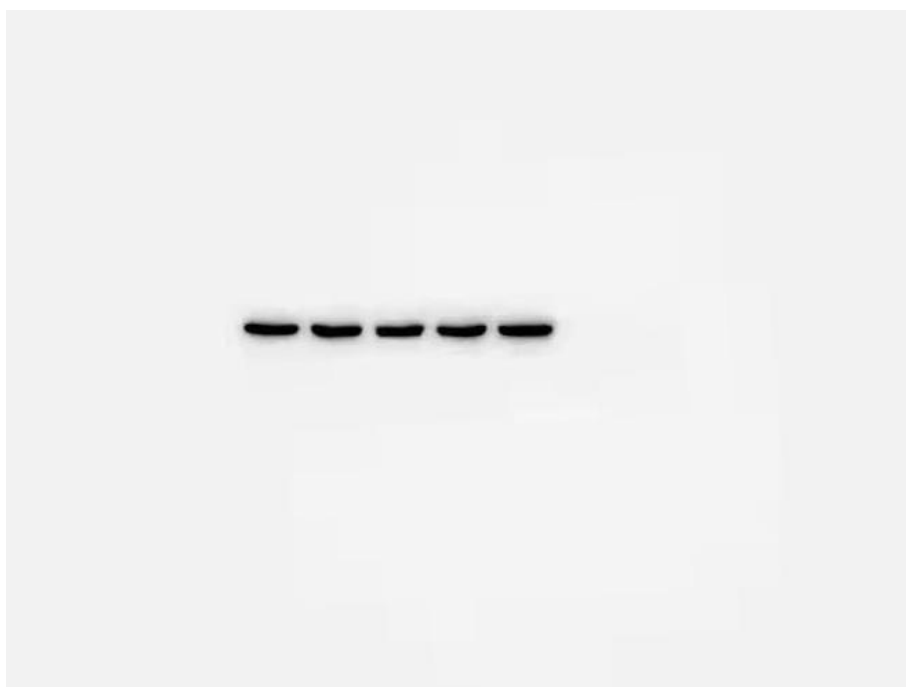

ADAMTS-5

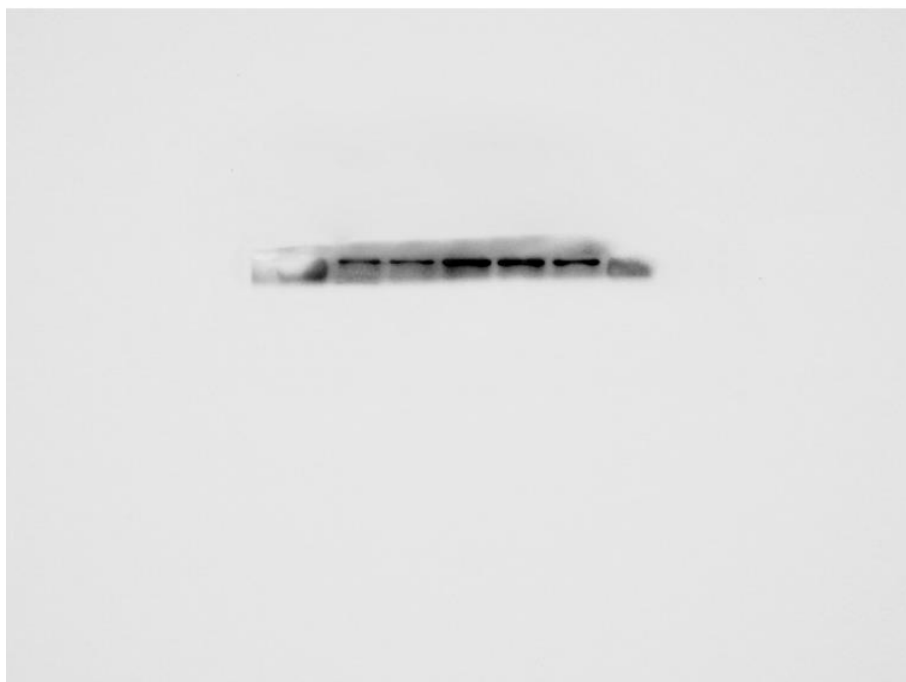

Aggrecan

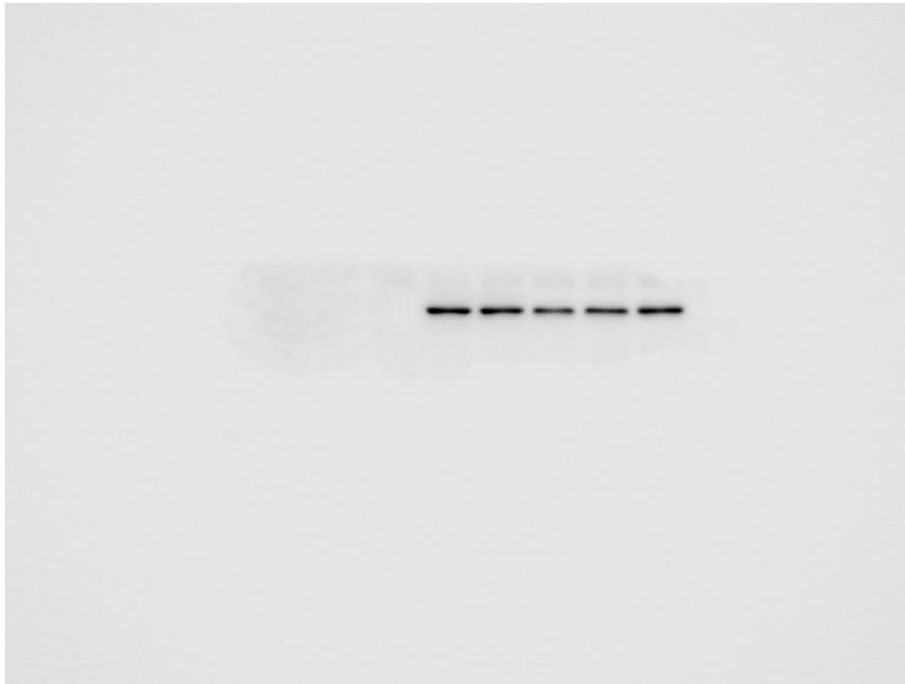

MMP-1

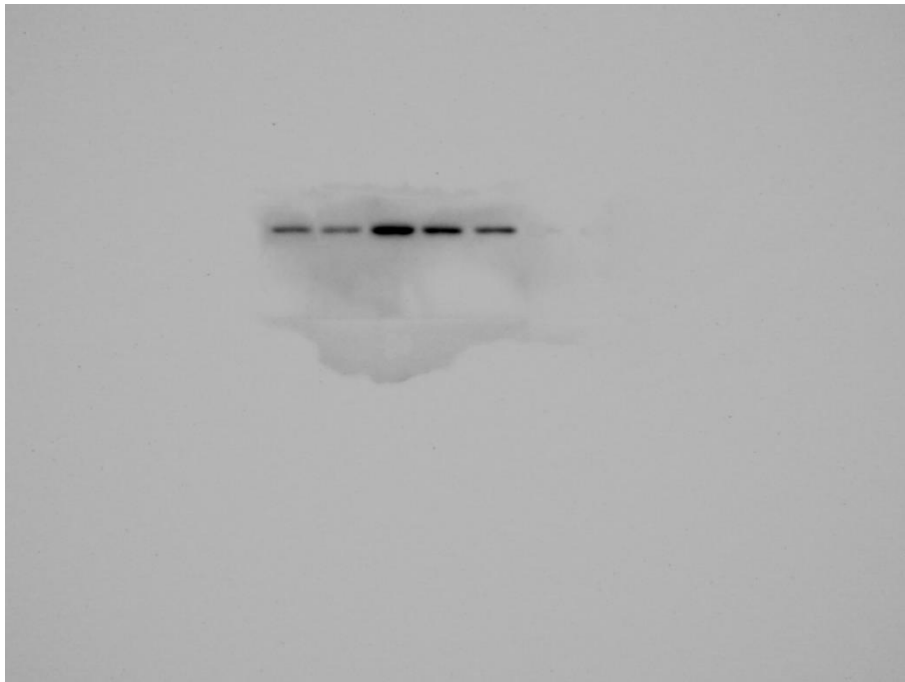

MMP-13

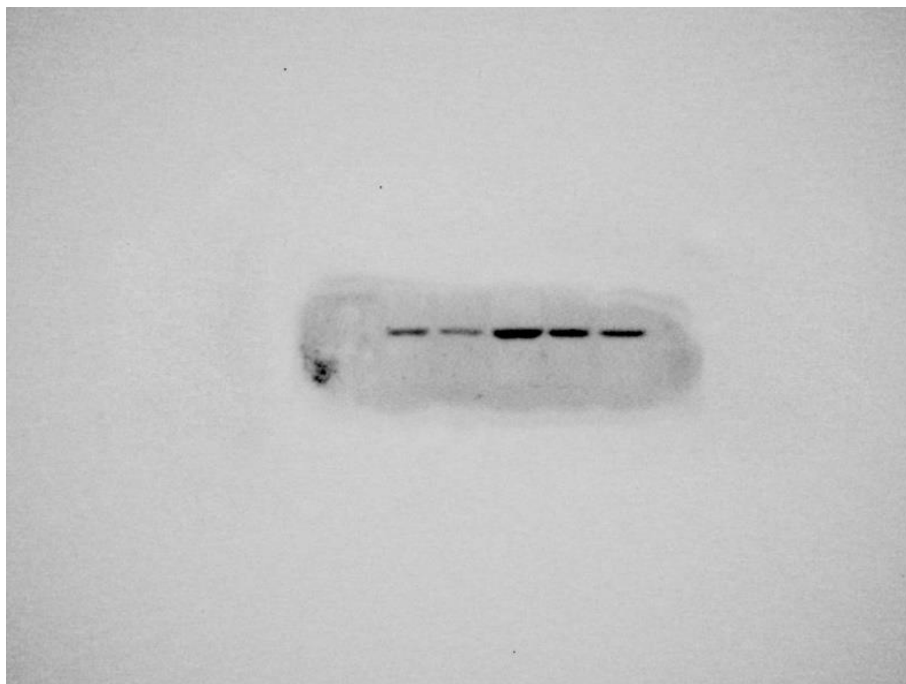

Actin

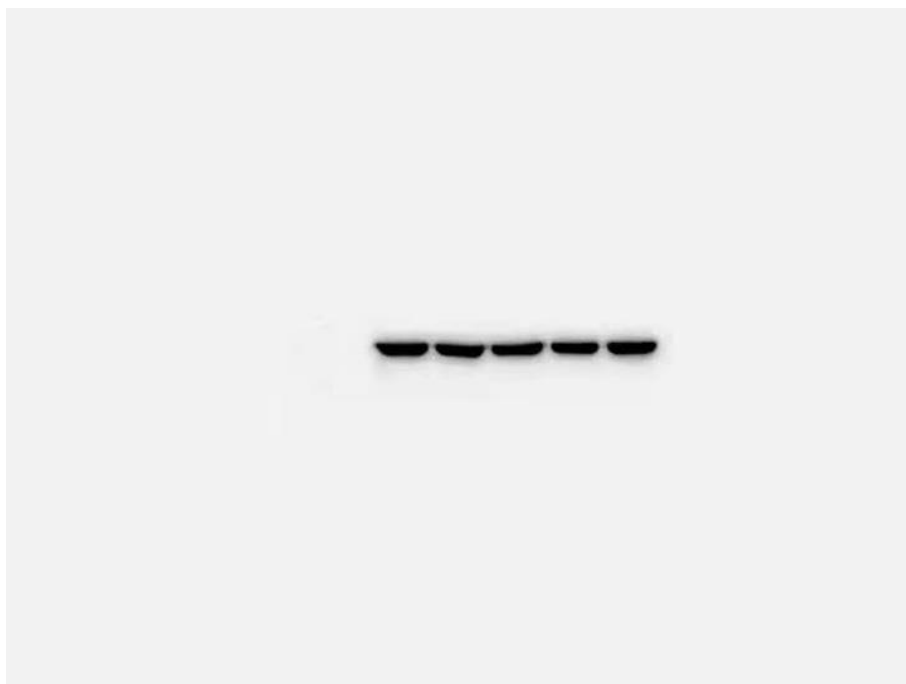

JNK

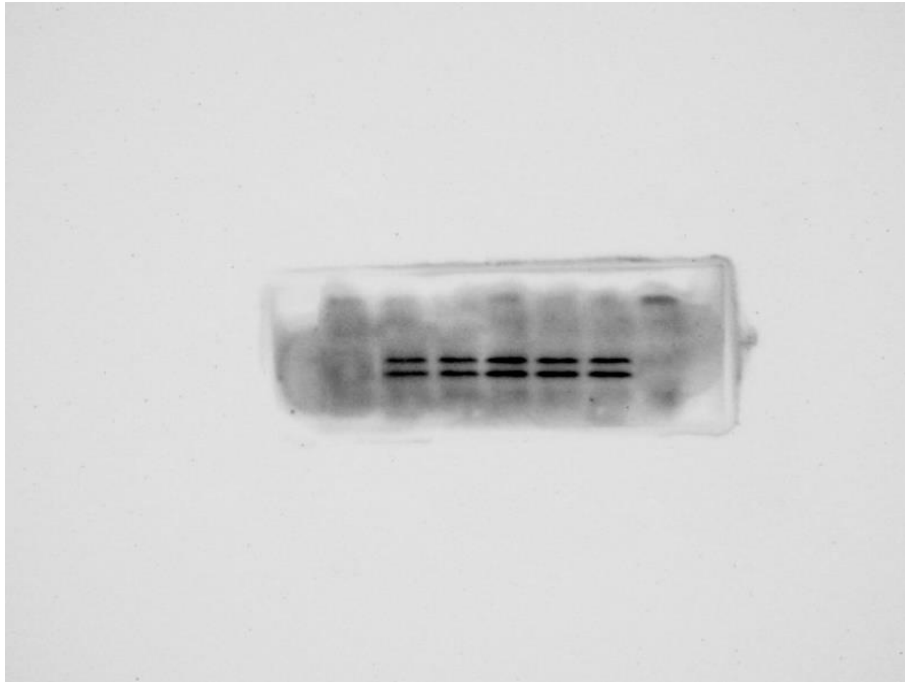

P-JNK

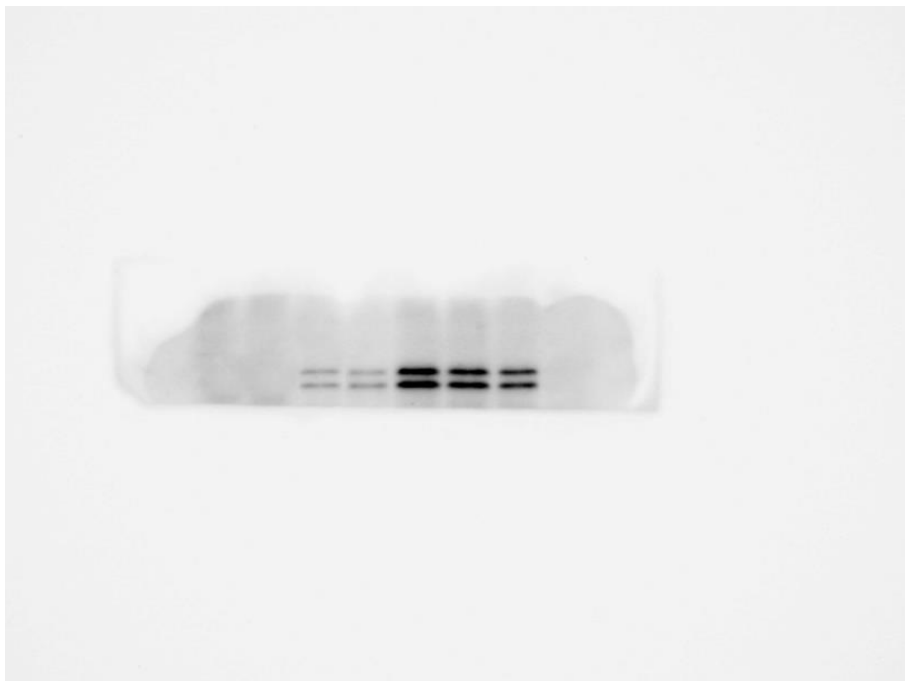

NF-KB

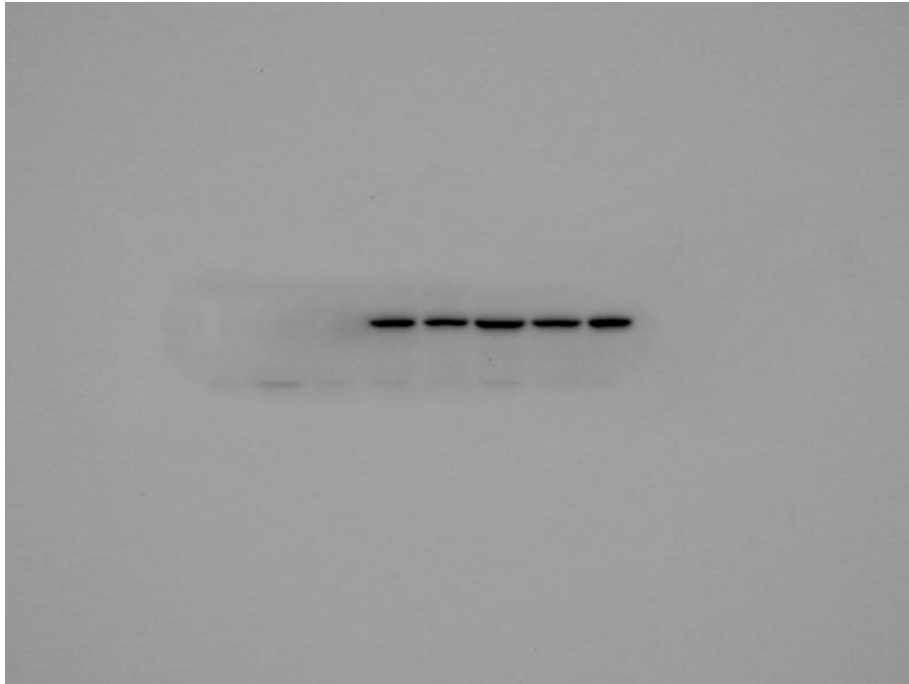

P-NF-KB

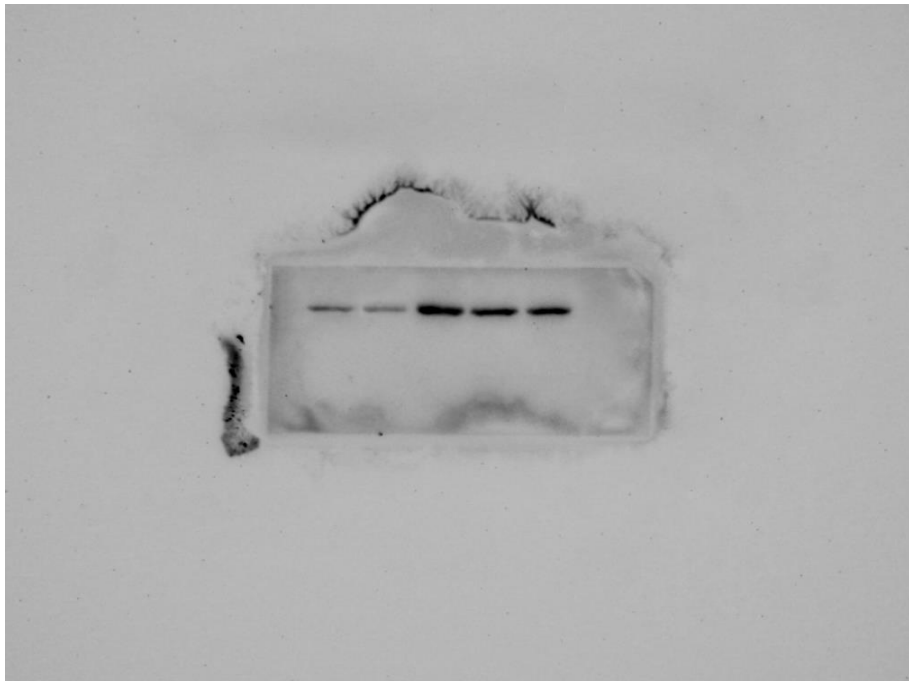

Supplement: Supplementary file 2 — Supplementary Information 2. [file 41598_2022_16093_MOESM2_ESM.pdf]

ADAMMTS 5

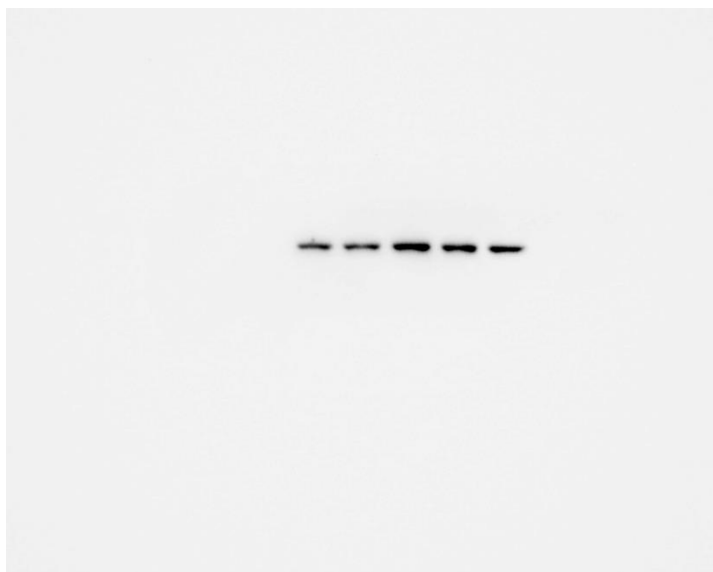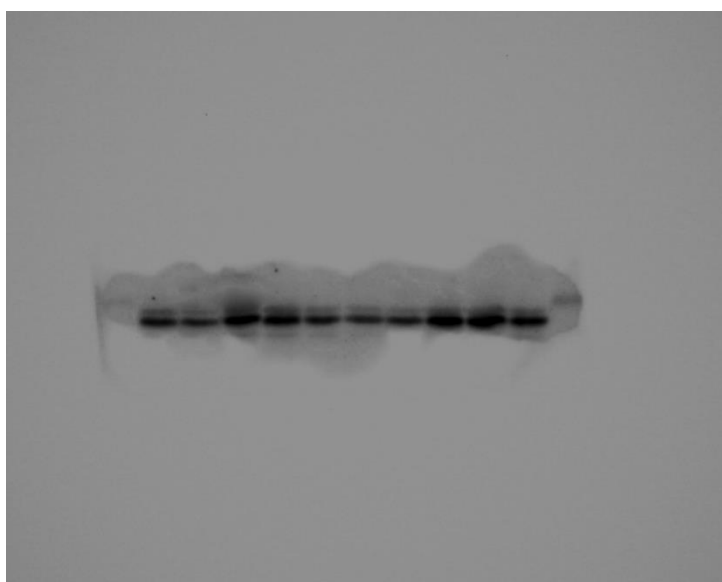

AggreCAN

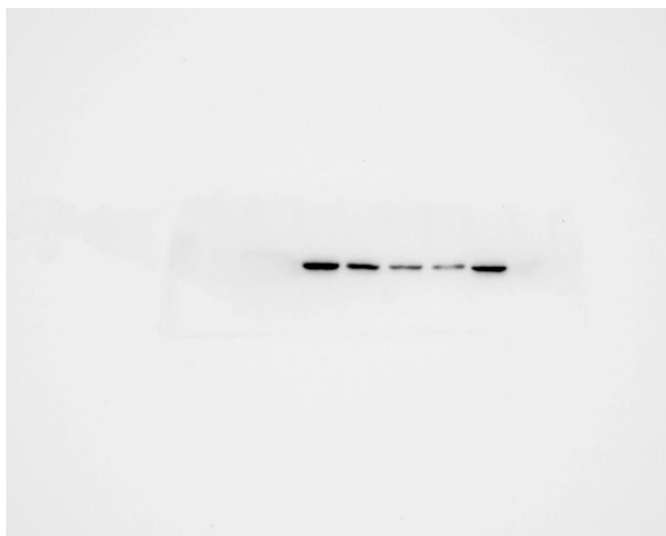

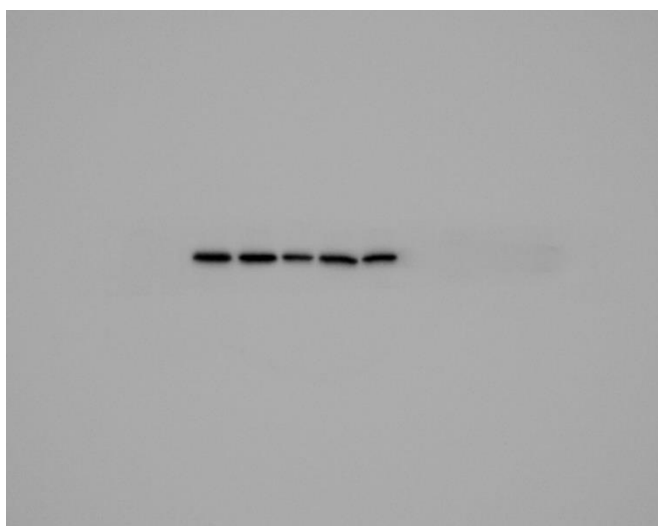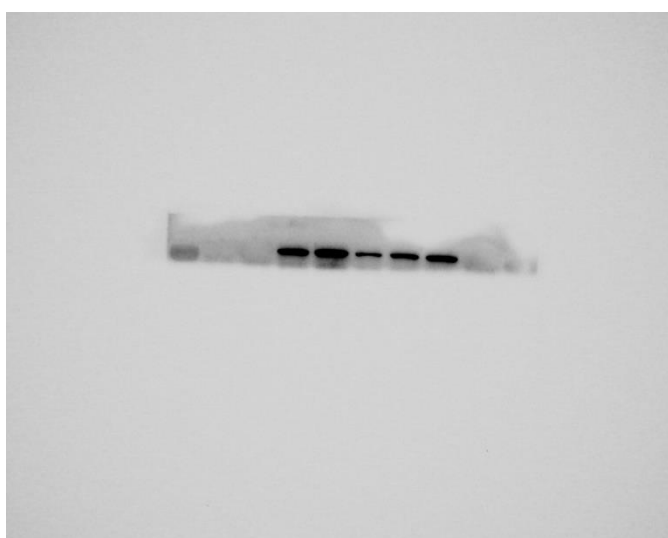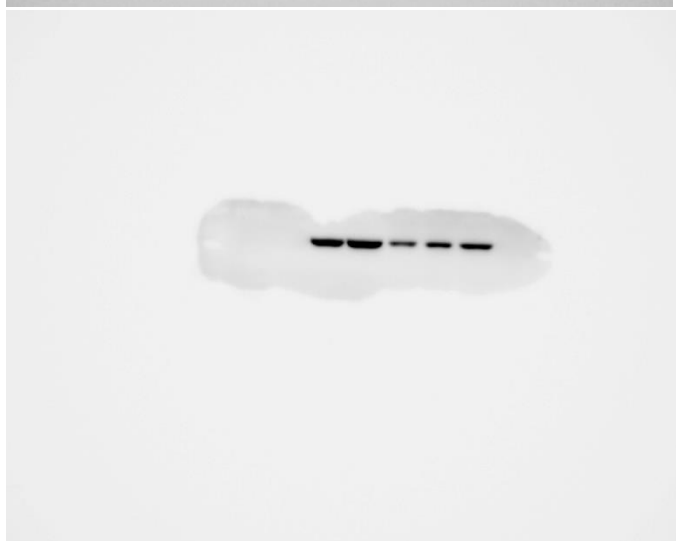

MMP-1

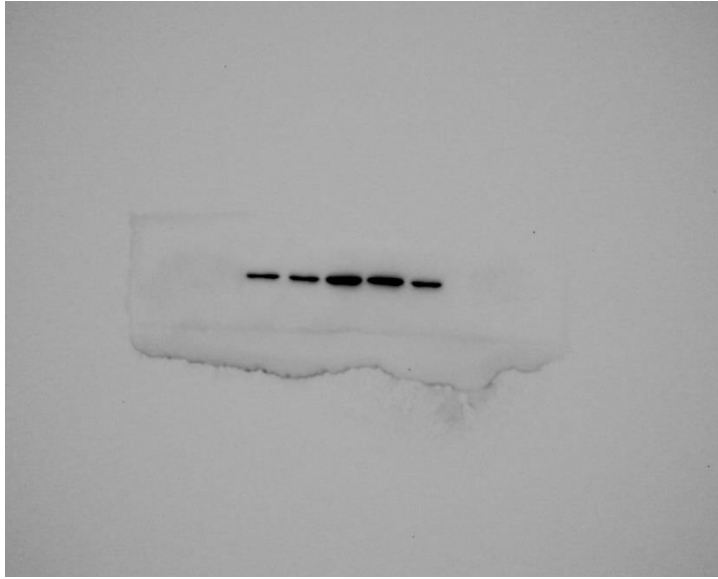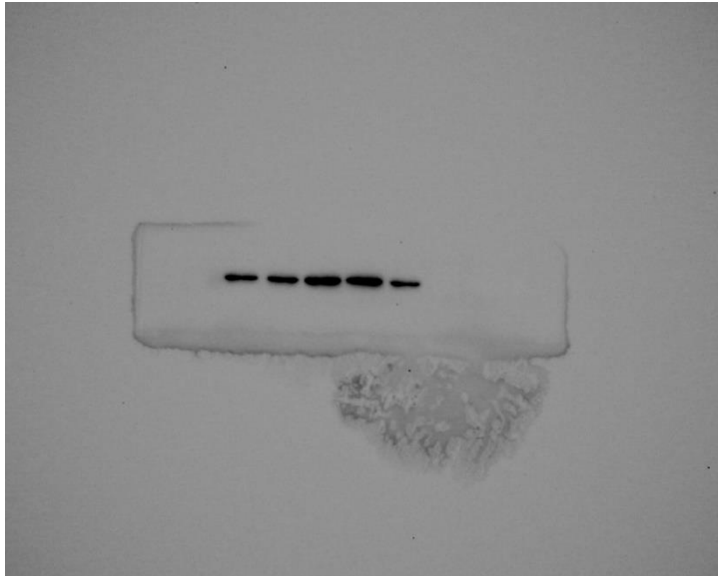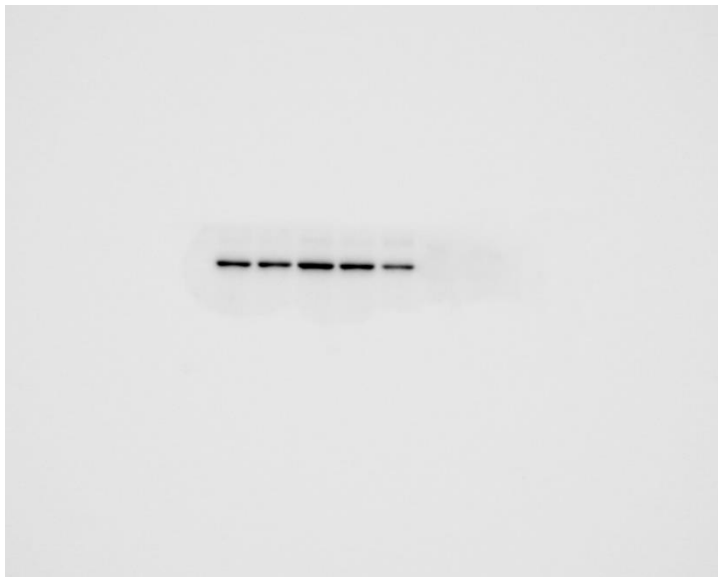

JNK

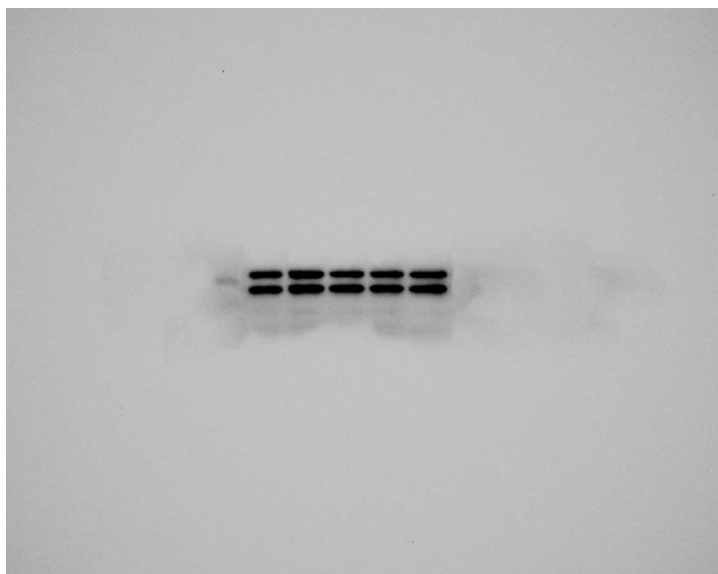

Mmp-13

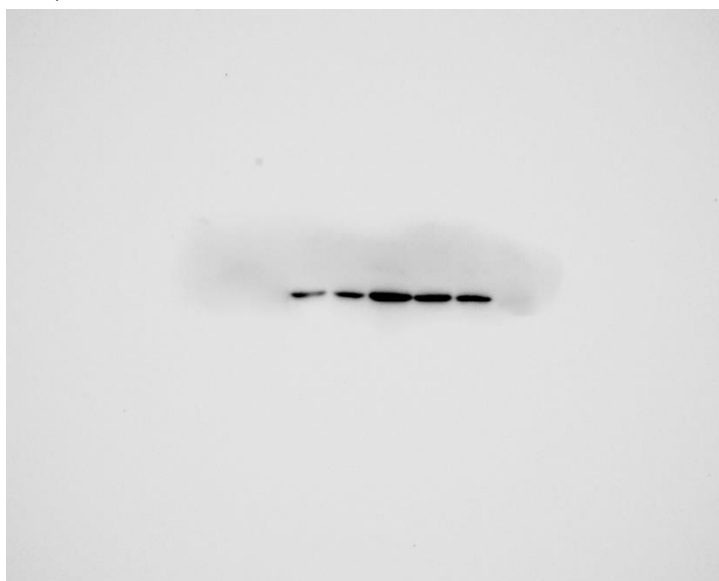

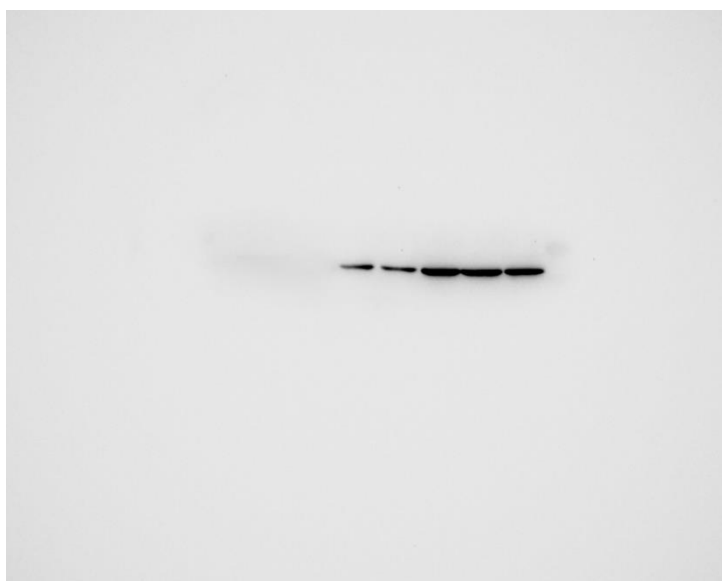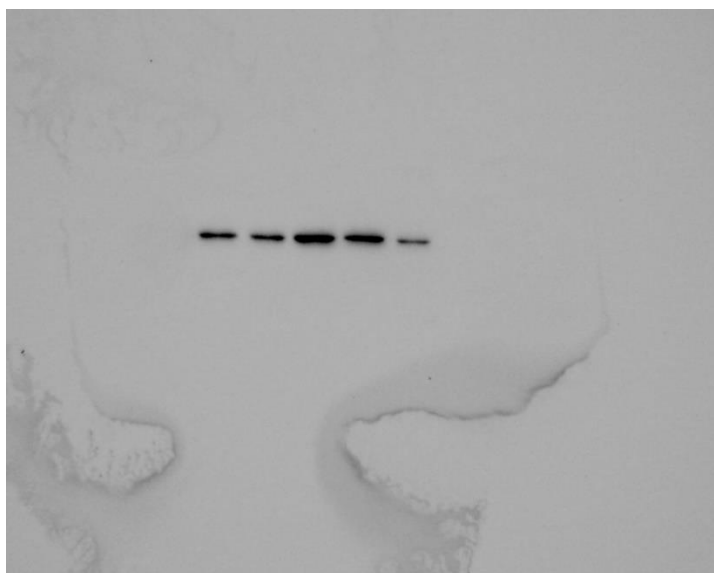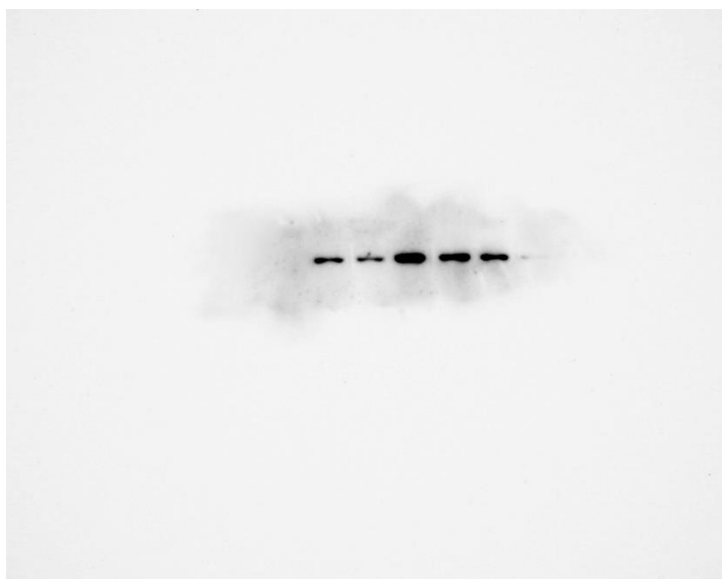

Pnfkb

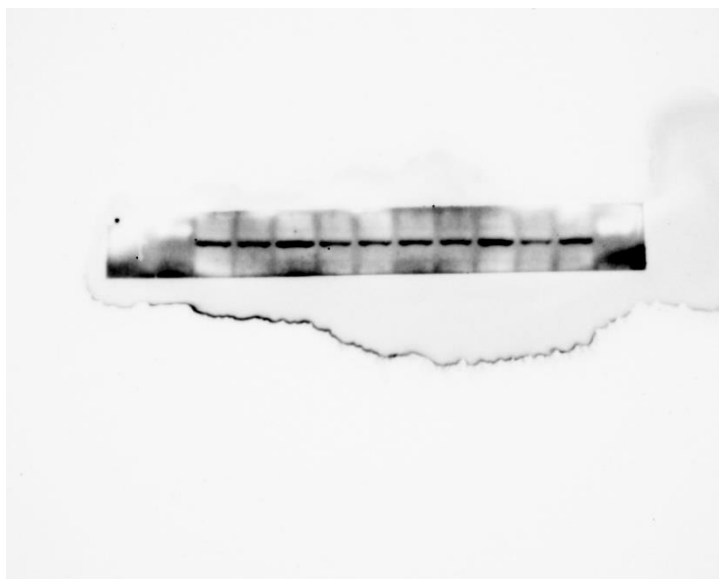

P-JNK

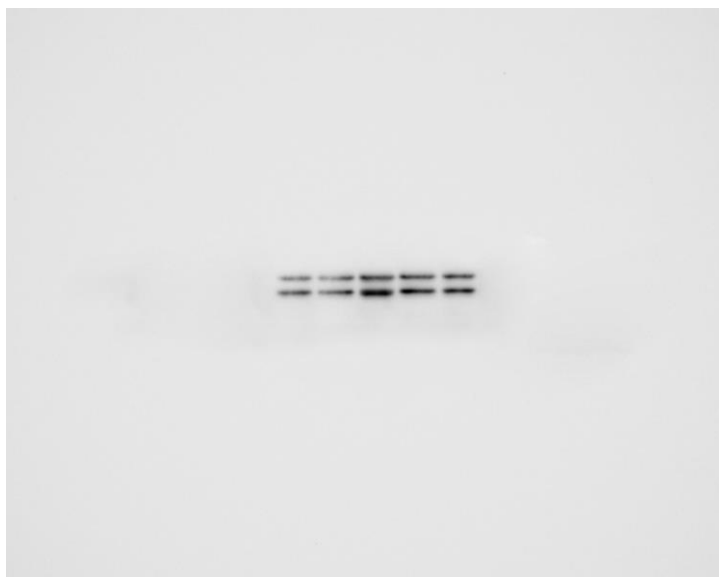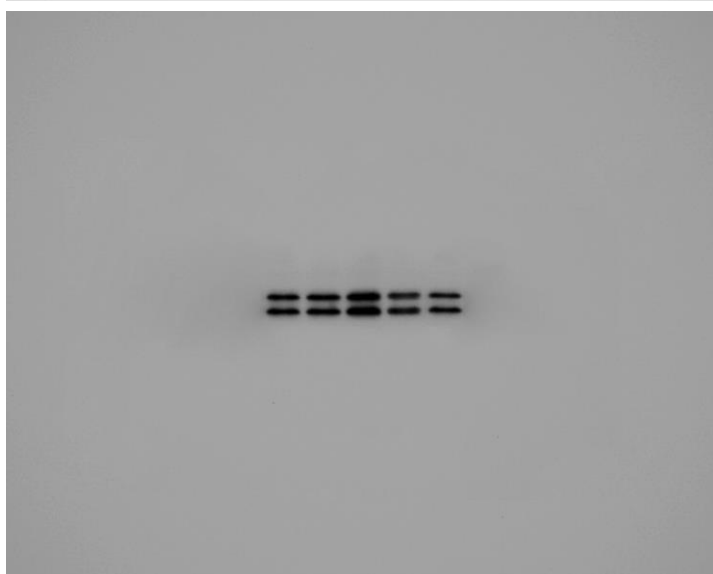

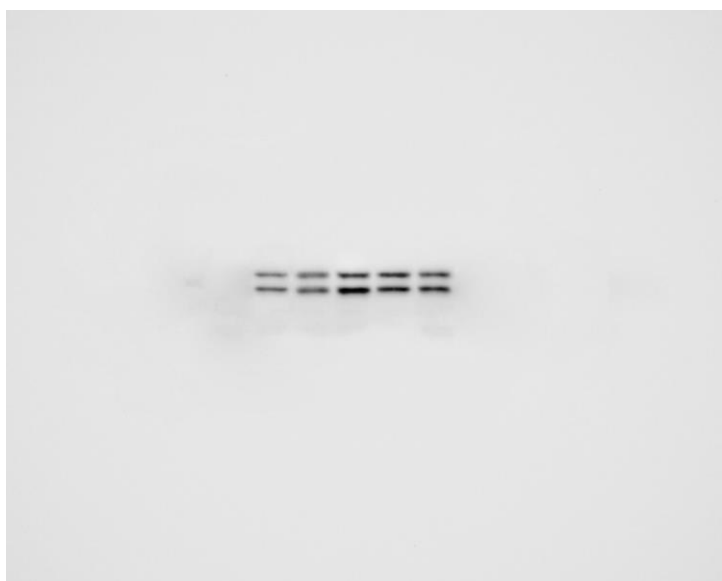

Supplement: Supplementary file 3 — Supplementary Information 3. [file 41598_2022_16093_MOESM3_ESM.pdf]
